# Membrane-bound TNF mediates microtubule-targeting chemotherapeutics-induced cancer cytolysis via juxtacrine inter-cancer-cell death signaling

**DOI:** 10.1038/s41418-019-0441-3

**Published:** 2019-10-23

**Authors:** Jing Zhang, Yu Yang, Shen’ao Zhou, Xueyan He, Xuan Cao, Chenlu Wu, Hong Hu, Jie Qin, Gang Wei, Huayi Wang, Suling Liu, Liming Sun

**Affiliations:** 1 State Key Laboratory of Cell Biology, CAS Center for Excellence in Molecular Cell Science, Shanghai Institute of Biochemistry and Cell Biology, Chinese Academy of Sciences, University of Chinese Academy of Sciences, 320 Yueyang Road, 200031 Shanghai, China; 2 Fudan University Shanghai Cancer Center & Institutes of Biomedical Sciences, Shanghai Medical College, Key Laboratory of Breast Cancer in Shanghai, Innovation Center for Cell Signaling Network, Cancer Institutes, Fudan University, 200032 Shanghai, China; 3 CAS Key Laboratory of Computational Biology, Collaborative Innovation Center for Genetics and Developmental Biology, CAS-MPG Partner Institute for Computational Biology, Shanghai Institute of Nutrition and Health, Shanghai Institutes for Biological Sciences, Chinese Academy of Sciences, 200031 Shanghai, China; 4 School of Life Science and Technology, Shanghai Tech University, 201210 Shanghai, China

**Keywords:** Cancer, Cell biology

## Abstract

Microtubule-targeting agents (MTAs) are a class of most widely used chemotherapeutics and their mechanism of action has long been assumed to be mitotic arrest of rapidly dividing tumor cells. In contrast to such notion, here we show—in many cancer cell types—MTAs function by triggering membrane TNF (memTNF)-mediated cancer-cell-to-cancer-cell killing, which differs greatly from other non-MTA cell-cycle-arresting agents. The killing is through programmed cell death (PCD), either in way of necroptosis when RIP3 kinase is expressed, or of apoptosis in its absence. Mechanistically, MTAs induce memTNF transcription via the JNK-cJun signaling pathway. With respect to chemotherapy regimens, our results establish that memTNF-mediated killing is significantly augmented by IAP antagonists (Smac mimetics) in a broad spectrum of cancer types, and with their effects most prominently manifested in patient-derived xenograft (PDX) models in which cell–cell contacts are highly reminiscent of human tumors. Therefore, our finding indicates that memTNF can serve as a marker for patient responsiveness, and Smac mimetics will be effective adjuvants for MTA chemotherapeutics. The present study reframes our fundamental biochemical understanding of how MTAs take advantage of the natural tight contact of tumor cells and utilize memTNF-mediated death signaling to induce the entire tumor regression.

## Introduction

Microtubule-targeting agents (MTAs), one of the most widely used chemotherapeutics, have gained great success in clinics over a long period [1]. Despite the well-validated role of MTAs in cancer treatment, the fundamental mechanistic question regarding MTA-induced cell death is still not clear. The clinical success of MTAs has been attributed to their ability to interfere with mitosis, resulting in so called ‘mitotic catastrophe’ and cancer cell death [2–4]. However, the clinical disappointment of non-MTA mitosis-specific inhibitors that specifically target the mitotic apparatus, such as mitotic kinases, and the fact that MTAs also kill non-dividing cancer cells, raise the possibility that the success of MTAs is perhaps not limited to impeding the completion of chromosome segregation in mitosis; MTAs may also target essential interphase cellular processes in cancer.

Much of our knowledge about the mechanism of MTAs inhibiting mitosis is exclusively based on the in vitro studies on fast-dividing cells in culture and xenograft models. In individual patients, and even at different locations within the same tumor, the cancer cell proliferation rates are very different [5], and marked mitotic arrest is rarely observed in patients post-MTA treatment [5, 6]. Accordingly, the mitotic arrest dogma cannot explain how a small dose of MTAs can not only target a small fraction of the tumor mass, where cells are actively dividing, but also reduce the mass of entire slowly growing tumors, such as breast cancers.

Tumor necrosis factor (TNF) is a member of a large family of cytokines that are able to induce the production of inflammatory cytokines, as well as induce cell death [7, 8]. TNF is first translated as a 26 kDa transmembrane molecule (memTNF) that is cleaved off the membrane by a metalloproteinase called TNF converting enzyme (TACE, also called ADAM17) to generate a soluble 17 kDa molecule (solTNF) [9, 10]. Both memTNF and solTNF are able to engage the TNF receptor 1 (TNFR1) to activate the downstream cell death pathways [11, 12]. In cells that do not express RIP3, or in which RIP3 signaling is defective, TNFR1 activates caspase-8, the initiator caspase, which subsequently cleaves and activates the downstream executioner caspase-3/7, resulting in apoptotic cell death [13, 14]. In cells that express functional RIP3 and have their caspase-8 inactivated, TNF family cytokines can trigger MLKL-dependent necroptosis [15–18]. Unlike other TNF superfamily members, such as FasL and CD40L, of which the membrane-bound and soluble forms often have different signaling potentials [19–24], the possible differences between memTNF and solTNF are poorly explored and still remain elusive. There are studies showing either opposing effect of the two forms of TNF [25] or identical responses of membrane mimic form and soluble form [26]. It is possible that memTNF and solTNF exert different stimulating capacities.

Here, we found a highly specific feature of MTA family drugs that differs from other mitosis-arresting agents: both microtubule depolymerizers and stabilizers can directly induce TNF-signaling-mediated tumor cell death, through either apoptosis in RIP3-dificient cancer cells or necroptosis in RIP3-expressing cancer cells. Surprisingly, this MTA-induced cancer cell death depends on JNK/c-Jun-regulated accumulation of membrane TNF (memTNF) but not on solTNF, through which to induce cancer-cell-to-cancer-cell killing. We also found that Smac mimetics, a group of small molecules working as inhibitors of the IAP (the inhibitor of apoptosis), can largely promote MTA-induced memTNF-mediated cancer cell death in vitro or cause PDX tumor regression in vivo. Our study indicates that memTNF accumulation can be monitored to track patient responsiveness and for optimizing both the clinical dosages of MTA drugs and their combination with other chemotherapeutics.

## Results

### MTAs induce necroptosis in L929 fibrosarcoma both in vitro and in vivo

**Fig. 1:**
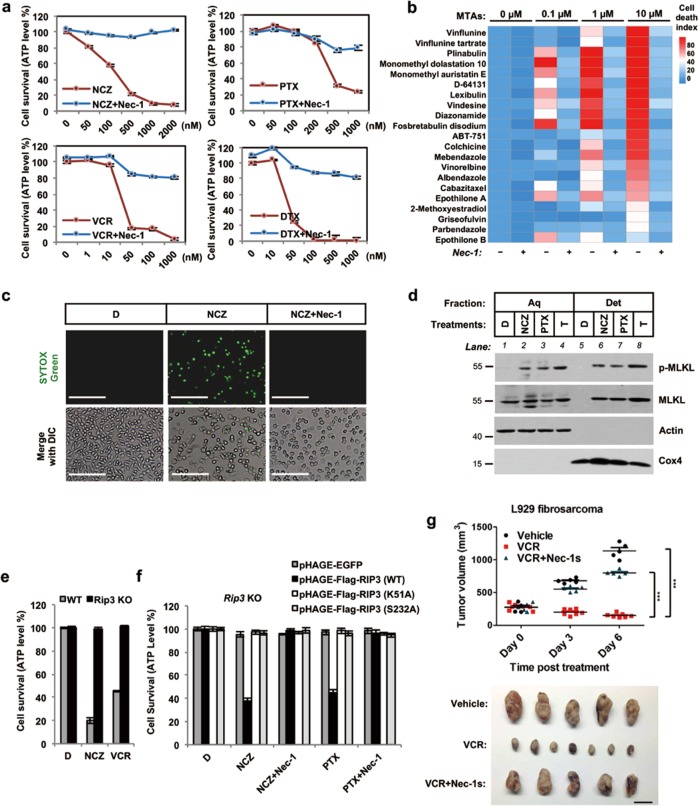
MTAs induce MLKL phosphorylation-dependent necroptosis in L929 fibrosarcoma, both in vitro and in vivo. **a** Dose-dependent necroptotic cytolysis effect of MTAs on L929 cells. **b** A panel of 21 MTAs was tested for necroptotic effect on L929 cells. Heat map analysis of cell death index was calculated based on ATP levels. **c** Fluorescent microscopy of SYTOX Green-labeled necroptotic L929 cells after NCZ treatment for 24 h. Plasma membrane breakdown was traced by SYTOX Green staining. Scale bar, 400 µm. **d** Immunoblotting analysis of MLKL phosphorylation by Triton X-114 fractionation in whole cell lysates of NCZ-treated or PTX-treated L929 cells. T, 20 ng/ml recombinant/soluble TNF treatment. Aq, aqueous fraction; Det, detergent fraction. **e** Effect of *Rip3* knockout on MTA-induced necroptosis in L929 cells. **f** Effect of RIP3 kinase activity on MTA-induced necroptosis in L929 cells. Wild-type or mutants of RIP3 were stably expressed in *Rip3* KO L929 cells by pHAGE infection. WT, wild-type RIP3; K51A, kinase dead form of RIP3; S232A, auto-phosphorylation site mutant of RIP3. RIP3 re-expression was detected by immunoblotting. **g** In vivo response of mouse allograft of L929 cells to VCR. Athymic nude mice bearing ~300 mm^3^ L929-fibrosarcoma were treated with vehicle or with 5 mg/kg Nec-1s and/or 5 mg/kg VCR. Upper: tumor growth was measured and calculated. Lower: representative image of L929 cells allografts on day 6. Vehicle, *n* = 5; VCR, *n* = 7; VCR + Nec-1s, *n* = 5. Scale bar, 1 cm. Graph shows mean ± SEM, *p* values were determined by the two-way ANOVA test; NS not significant; *p < 0.05; **p < 0.01; ***p < 0.001. D, DMSO; NCZ, nocodazole; VCR, vincristine; PTX, paclitaxel; DTX, docetaxel. Cell viability was determined by measuring ATP levels. The data are represented as mean ± SEM of duplicate wells (**a**, **b**, **e**, and **f**). Results are reported from one representative experiment. Experiments were repeated independently for four (**a**, **c**), three (**d**–**g**), or two (**b**) times

L929 is a mouse fibrosarcoma cell line that shows exceptional sensitivity to TNF-mediated programmed necrosis, also called necroptosis [27]. We used L929 cells to perform a large-scale compound screening, and found several MTAs, including both microtubule depolymerizers like nocodazole (NCZ) and vincristine (VCR), as well as microtubule stabilizers such as paclitaxel (PTX) and docetaxel (DTX), that directly induced cell death in a dose-dependent manner (Fig. 1a). The nature of this form of cell death was apparently necroptosis, since it can be blocked by a necroptosis-specific RIP1 kinase inhibitor Nec-1 [28] (Fig. 1a). In addition, expression of the kinase-dead form of RIP1 (K45A) in *Rip1* knockout L929 cells completely abrogated MTA-induced cell death (Supplementary Fig. 1a–d).

To see if the observed necroptosis induced by MTAs is a general effect of MTA treatment, we tested a large collection of MTAs, and found that they inevitably caused Nec-1-blockable cell death in a dose-dependent-manner (Fig. 1b). Then we tested several different classes of cell-cycle-arresting agents, but none showed the necroptosis-inducing effect as MTAs did (Supplementary Fig. 2), indicating that necroptosis induction in L929 cells is apparently restricted to MTAs.

A classical morphological change of necroptotic cells is the loss of plasma membrane integrity [29]. Staining with SYTOX Green, a cell-impermeable nucleic acid dye, we observed that a large population of NCZ-treated L929 cells lost their cellular integrity, and this process could be blocked by Nec-1 (Fig. 1c and Supplementary Fig. 1e, f). In addition, we detected (i) MLKL phosphorylation and (ii) its presence in membrane fraction—two hallmarks of necroptosis [15, 30]—in a time-dependent and dose-dependent manner (Fig. 1d and Supplementary Fig. 3a–d). Consistently, knocking out *Mlkl* completely blocked this form of MTA-induced necroptosis (Supplementary Fig. 3e–h). We also found that *Rip3* knockout or ectopic expression of either the kinase-dead form (RIP3-K51A) or the auto-phosphorylation site mutant (RIP3-S232A) blocked MTA-induced necroptosis (Fig. 1e, f and Supplementary Fig. 3i–k). Taken together, our results establish that MTA-induced necroptosis in L929 cells depends on the classical RIP1–RIP3–MLKL pathway.

We subsequently tested whether MTA treatment leads to RIP1-mediated necroptosis in vivo using the mouse L929 fibrosarcoma allograft model in nude (athymic) mice [31, 32]. Similar to our in vitro findings, MTA treatment (here we used VCR) led to a significant tumor regression, and co-treatment with Nec-1s blocked this VCR-induced L929 tumor regression (Fig. 1g).

### MTAs promote cancer cell juxtacrine cytotoxic membrane-bound TNF

**Fig. 2:**
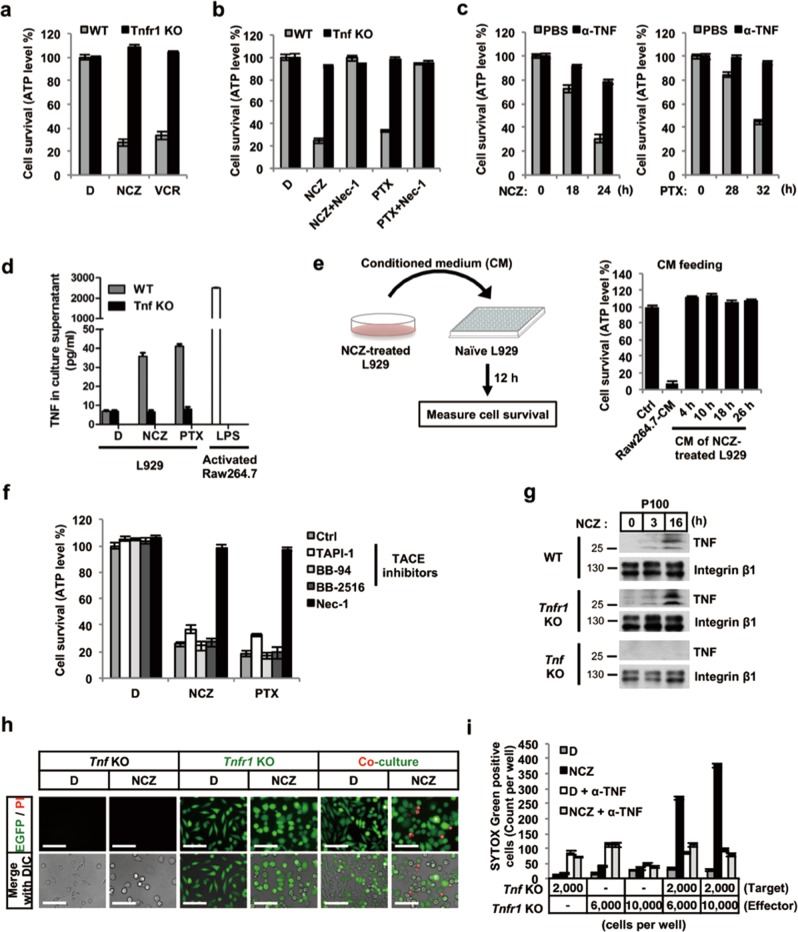
MTAs activate membrane TNF signaling to induce bystander cell death. **a**, **b** Effect of *Tnfr1* (**a**) and *Tnf* (**b**) knockout on MTA-induced necroptosis in L929 cells. **c** Pretreatment (2 h) of neutralizing antibody against TNF rescued cells from MTA-induced necroptosis. **d** MTA-treated L929 cells were tested for the presence of soluble TNF (solTNF) in the cell culture media. Samples were harvested for ELISA analysis to determine the concentration of solTNF, as described in the “Methods” section. LPS-primed Raw264.7 cell medium was used as a positive control for measuring the autocrined soluble TNF. **e** MTA-treated L929 conditioned medium (CM) was applied to naïve cells. Left panel, a schematic representation of the experimental design. Right panel, conditioned medium-fed L929 cell viability was determined by ATP levels at 12 h post treatment. **f** Influence of TACE inhibitors on MTA-induced cell death in L929 cells. TACE inhibitors were pretreated for 2 h followed by MTAs treatment. **g** Immunoblotting analysis of membrane-bound TNF in crude membrane fraction of NCZ-treated WT, *Tnfr1* KO, and *Tnf* KO L929 cells. Integrin β1 was used as the loading control of plasma membrane fraction. **h**, **i** MTA-induced bystander cell death analysis in L929 *Tnf* KO and *Tnfr1* KO co-culture system, as described in the “Methods” section. Representative images show NCZ-induced necroptotic cells by PI staining (**h)**. Scale bar, 100 µm. The number of necroptotic cells per well was quantified by IncuCyte (**i)**. D, DMSO; NCZ, nocodazole; VCR, vincristine; PTX, paclitaxel. Cell viability was determined by measuring ATP levels. The data are represented as mean ± SEM of duplicate wells (**a**–**f** and **i)**. Results are reported from one representative experiment. Experiments were repeated independently for four (**a**, **b**, and **g**) or three (**c**–**f**, **h,** and **i**) times

To further investigate the death signal initiation of MTA-induced necroptosis, firstly, we found that MTA-induced necroptosis was completely blocked in the *Tnfr1* knockout L929 cells and that this cell death phenotype could be rescued via re-expression of TNFR1 (Fig. 2a and Supplementary Fig. 4a–c). Similarly, MTA-induced necroptosis was abolished in the *Tnf* knockout L929 cells (Fig. 2b and Supplementary Fig. 4d). Further, by using antisera that neutralizes TNF activity, we found that MTA-induced necroptosis was prevented in L929 cells (Fig. 2c). These results demonstrated MTA-induced necroptosis in L929 cells is initiated by TNFR1 activation.

Upon MTA treatment, the secreted TNF in the L929 culture medium was slightly increased, while the absolute soluble TNF (solTNF) levels were negligible compared with those in activated macrophage cell line Raw264.7 medium (Fig. 2d). Raw264.7 and L929 cells were subjected to LPS and MTA treatments, respectively (Fig. 2e and Supplementary Fig. 4e). We found that naïve L929 cells were highly susceptible to the lytic effect of Raw264.7-conditioned medium (CM) (Supplementary Fig. 4e), whereas they were completely resistant to NCZ-treated L929-CM. These results indicated that, unlike Raw264.7 macrophages, the amount of cell-free TNF secreted by MTA-treated L929 cells was insufficient to induce necroptosis in the target cells.

We further confirmed this notion by utilizing TACE inhibitors to block solTNF shedding and found that MTA-induced L929 necroptosis was not affected (Fig. 2f and Supplementary Fig. 4f), whereas the Raw264.7-CM-induced necroptosis was abrogated (Supplementary Fig. 4e, f). Clearly, the necroptosis observed in MTA-treated L929 cells was not due to autocrine secretion of TNF. Therefore, we speculated that it may be the memTNF that contributed to MTA-induced killing.

To explore this possibility, after MTA treatment, we separated the cytosolic and membrane fractions and found that memTNF accumulated in the P100 fraction, which represents the plasma membrane compartment: both wild-type (WT) and *Tnfr1* knockout cells showed a similar level of memTNF accumulation, while *Tnf* knockout cells did not (Fig. 2g and Supplementary Fig. 4g, h). We also tested several other groups of cell-cycle-arresting agents and found that none of them caused memTNF accumulation (Supplementary Fig. 4i, j).

To confirm that memTNF-mediated killing depends on cell–cell contact, we co-cultured *Tnfr1* knockout L929 cells that stably expressed EGFP with *Tnf* knockout L929 cells. Although both of the *Tnfr1* and *Tnf* knockout L929 cells were completely resistant to MTA-induced necroptosis (Fig. 2a, b, and Supplementary Fig. 4c, d), when these cells were co-cultured together, the *Tnfr1* knockout L929 cells remained alive after NCZ treatment, while the *Tnf* knockout L929 cells (non-GFP labeled) were cytolysed, and the extent of this cell death increased as the ratio of *Tnfr1* knockout cells was increased. In addition, this trans-signaling (between cells) induced cell death was blocked by the anti-TNF neutralizing antiserum (Fig. 2h, i). These results from our co-culture system supported the idea of memTNF as the principal inducer of bystander cancer cell death. In accordance with this finding, we found that MTA-induced necroptosis in L929 is cell density dependent (Supplementary Fig. 4k).

### MTAs activate JNK/c-Jun to promote TNF transcription and memTNF accumulation

**Fig. 3:**
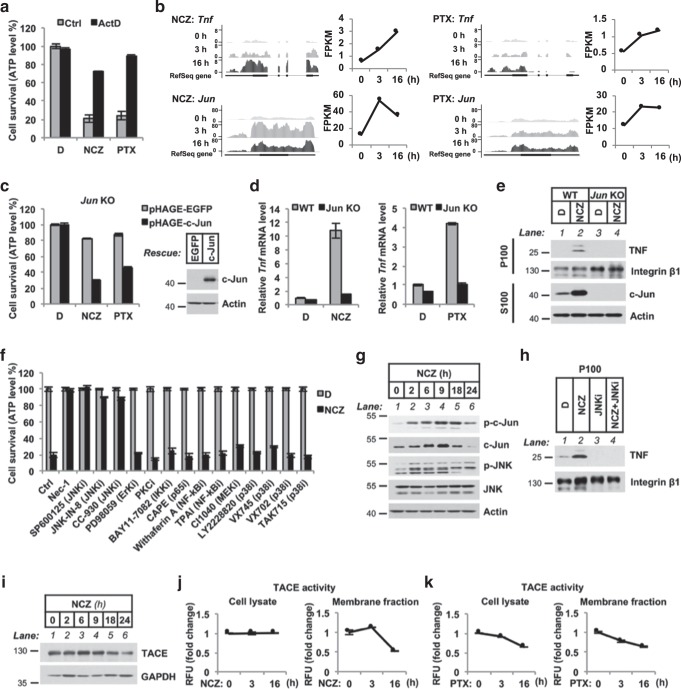
MTAs upregulate membrane TNF transcription through the JNK/c-Jun axis. **a** Effect of transcriptional inhibitor actinomycin D (ActD) on MTA-induced cell death in L929 cells. **b** RNA-sequencing analysis of *Tnf* and *Jun* gene expression patterns during MTA treatment. UCSC genome browser images depict calculated FPKM (fragments per kilobase of transcript per million mapped reads) values in RNA-sequencing data. Gene expression levels are provided in DATA SET  1. **c** Effect of *Jun* knockout on MTA-induced necroptosis in L929 cells. For complementation, wild-type c-Jun was expressed in the knockout cells and its expression was detected by immunoblotting. **d** qRT-PCR analysis of *Tnf* mRNA level in MTA-treated WT and *Jun* KO L929 cells. **e** Immunoblotting analysis of TNF in P100 fractions of NCZ-treated WT and *Jun* KO L929 cells. **f** A panel of MAPK and NF-κB inhibitors was tested for necroptosis inhibition effect on MTA-treated L929 cells. All inhibitors were pretreated for 2 h followed by NCZ challenge. i, inhibitor. **g** Immunoblotting analysis of the JNK/c-Jun activation in whole cell lysates of NCZ-treated L929 cells. **h** Immunoblotting analysis of TNF accumulation in membrane fraction (P100) in the presence of JNK inhibitor (JNKi, SP600125) for NCZ-treated L929 cells. **i** Immunoblotting analysis of TACE expression in whole cell lysates of NCZ-treated L929 cells. **j**, **k** Fluorimetric assay of measuring TACE activity in both cell lysate (left panel) and membrane fraction (right panel) of NCZ (**j**) or PTX (**k**) treated L929 cells. D, DMSO; NCZ, nocodazole; PTX, paclitaxel. Cell viability was determined by measuring ATP levels. The data are represented as mean ± SEM of duplicate wells (**a**, **c**, and **f**). Results are reported from one representative experiment. Experiments were repeated independently for four (**f**), three (**a**, **c**, **d**, and **g**), or two (**e** and **h**–**k**) times

To explore the mechanism by which MTAs regulate TNF activation, We found that the transcriptional inhibitor actinomycin D (ActD) completely blocked both NCZ-induced and PTX-induced cell death (Fig. 3a), indicating that MTAs activated *Tnf* gene transcription. Then we performed RNA sequencing analysis on MTA-treated L929 cells (Fig. 3b), and observed that NCZ or PTX treatment increased the endogenous *Tnf* mRNA level in a time-dependent manner. We performed cluster analysis using the RNA-sequencing count data for all 1477 annotated transcription factors in the mouse genome. Notably, one of the clusters comprised the set of genes that respond quickly upon MTA treatment, peaking at 3 h and then falling at 16 h, earlier than the *Tnf* mRNA response (Supplementary Fig. 5a, b; see the entire list of genes in DATA SET 1). A literature search revealed that, among the transcription factors of this cluster, NF-κB and c-Jun were reported to be associated with *TNF* transcription [33, 34]; but there are no reports about them in cancer cells in response to MTAs. Firstly, we knocked down the genes for *NF-κB* transcription factors *Rela* and *Relb*, which regulate canonical and non-canonical NF-κB pathway, respectively, and found there was no effect on MTA-induced cell death or *Tnf* mRNA upregulation (Supplementary Fig. 5c–f). We then generated L929 *Jun* knockout cell lines and, and found the MTA-induced necroptosis was abolished; combined with the rescue of c-Jun expression, cell death was recovered (Fig. 3c). Additionally, *Tnf* mRNA and protein upregulation—as well as memTNF accumulation—were all abolished by *Jun* knockout (Fig. 3d, e).

We further screened a panel of kinase inhibitors of the MAPK pathways to look for the upstream kinase of c-Jun. We found that JNK inhibitors could fully block NCZ-induced cell death (Fig. 3f). Immunoblotting of NCZ-treated L929 cells showed increased c-Jun expression and enhanced JNK activation (Fig. 3g). Further, NCZ-induced memTNF accumulation also vanished after pretreatment with the JNK inhibitor SP600125 (Fig. 3h). Previous reports have shown JNK regulate solTNF autocrine production in additional contexts [35–37]. Our results revealed a specific and novel role of JNK kinase for MTA-regulated memTNF transcription.

Next, we analyzed the transcriptional effect of other non-MTA cell-cycle-arresting agents in L929 cells. qPCR results showed *Jun* and *Tnf* mRNA levels stayed constant (Supplementary Fig. 5g–i). Neither c-Jun nor JNK was activated in response to these agents, and their expression levels did not change throughout the treatment (Supplementary Fig. 5j–l).

Although the TACE level is normally stable, iRhom2, an ER protein, that is upregulated to activate TACE when memTNF must be cleaved into solTNF [38, 39]. Based on the RNA-sequencing data, we found that neither the *Tace* nor *Rhbdf2* (the gene that encodes iRhom2) mRNA level changed upon MTA treatment (Supplementary Fig. 5m). We also observed that the neither the TACE protein level nor the TACE activity increase upon MTA treatment (Fig. 3i–k). Together, these results explained how TNF transcription is elevated in response to MTAs while little solTNF is shed outside of the cells.

### MTAs induce memTNF-mediated apoptosis in RIP3-deficient human carcinoma cell lines

**Fig. 4:**
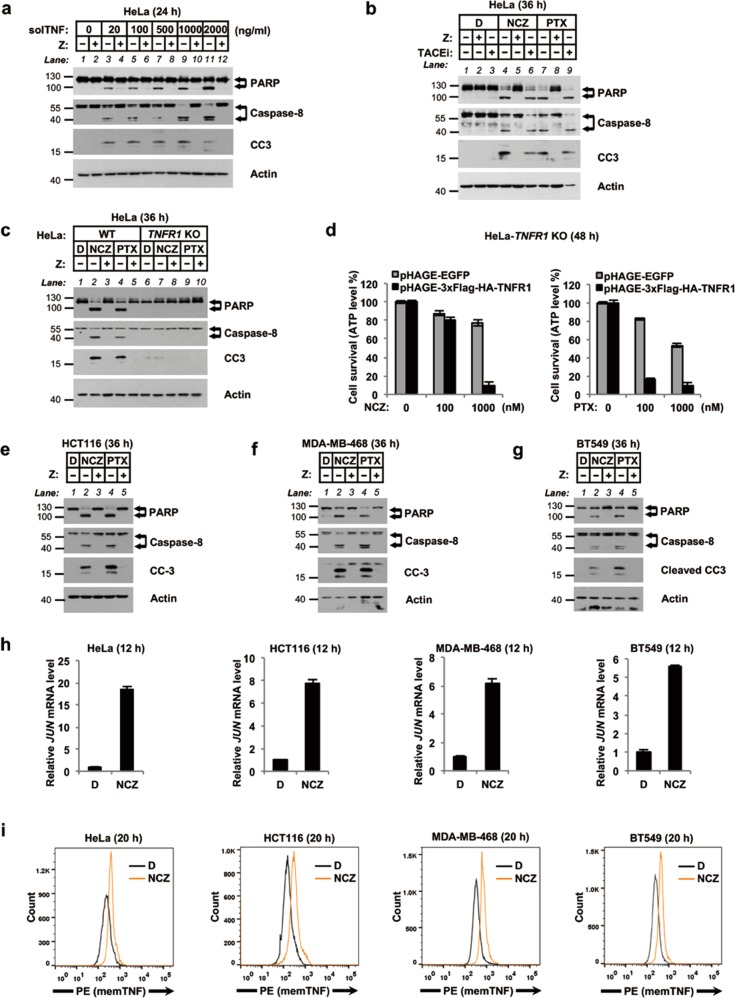
MTAs induce memTNF-mediated apoptosis in RIP3-deficient human carcinoma cell lines. **a** Immunoblotting analysis of apoptosis markers using whole cell lysates from recombinant TNF (soluble TNF, solTNF)-treated HeLa cells in the presence or absence of pan-caspase inhibitor z-VAD (Z). Cells were treated as indicated for 24 h. **b** Immunoblotting analysis of apoptosis markers using whole cell lysates from 1 µM MTA-treated HeLa cells in the presence or absence of 20 µM TACE inhibitor TAPI-1 (TACEi) or 20 µM pan-caspase inhibitor z-VAD (Z) for 36 h. **c** Immunoblotting analysis of apoptosis markers using whole cell lysates from 1 µM MTA-treated WT and *TNFR1* KO HeLa cells for 36 h. **d** Effect of *TNFR1* knockout on MTA-induced cell death in HeLa cells. Cells were treated as indicated for 48 h. **e**–**g** Immunoblotting analysis of apoptosis markers using whole cell lysates of 1 µM MTA-treated HCT116 (colon cancer, **e**), MDA-MB-468 (breast cancer, **f**), and BT549 (breast cancer, **g**) cells for 36 h. **h**, **i** qRT-PCR analysis of *JUN* mRNA level (**h**) and in flow cytometric analysis of memTNF (**i**) in MTA-treated HeLa, HCT116, MDA-MB-468, and BT549 cells for 12 and 20 h respectively. D, DMSO; NCZ, nocodazole; PTX, paclitaxel; Z, z-VAD. Cell viability was determined by measuring ATP levels. The data are represented as mean ± SEM of duplicate wells (**d**). Results are reported from one representative experiment. Experiments were repeated independently three (**c**, **d**, and **h**) or two (**a**, **b**, **e**–**g**, and **i**) times

A large proportion of human cancer cells, such as HeLa cells, are resistant to necroptosis but sensitive to apoptosis, due to lack of expression of necroptosis essential gene *RIP3* [40–43]. We next tested the cell death responsiveness to MTAs of RIP3-deficient human cancer cell lines to extend our study beyond necroptosis-sensitive cells. We found MTAs can also induce TNF-dependent apoptosis in HeLa cells; blocking soluble TNF shedding by inhibiting TACE activity did not tamper with MTA-induced apoptosis; cell death was abolished when *TNFR1* was knocked out (Fig. 4a–c); ectopic expression of TNFR1 in *TNFR1* knockout HeLa cells restored the apoptotic response to MTAs (Fig. 4d and Supplementary Fig. 6a, b). We next confirmed MTA-induced apoptosis in multiple RIP3-dificient human cancer cell lines (Fig. 4e–g). And indeed, the mRNA levels of *JUN* and the membrane-bound TNF of these human cancer cells were also upregulated in response to treatment with MTAs (Fig. 4h, i).

### MTA-induced memTNF-mediated apoptosis can be potentiated by antagonizing IAP activity with Smac mimetics

It was noticed that MTA-alone-induced apoptosis largely relies upon high dosage and long-time treatment, which inspired us next focused on optimization of MTAs in chemotherapy regimens. Given that IAP proteins block apoptosis caused by TNF, and considering that IAP antagonists have profound effects in potentiating solTNF-induced apoptosis [44–46], we hypothesized a combination treatment with IAP antagonists Smac mimetics (S) would further sensitize cancer cells to MTA-induced apoptosis. Currently, several Smac mimetics are being evaluated in clinical trials (https://clinicaltrials.gov) and combination of LCL161 with PTX has shown synergistic effects on several cancers [47–49], but the mechanism remains largely unknown.

**Fig. 5:**
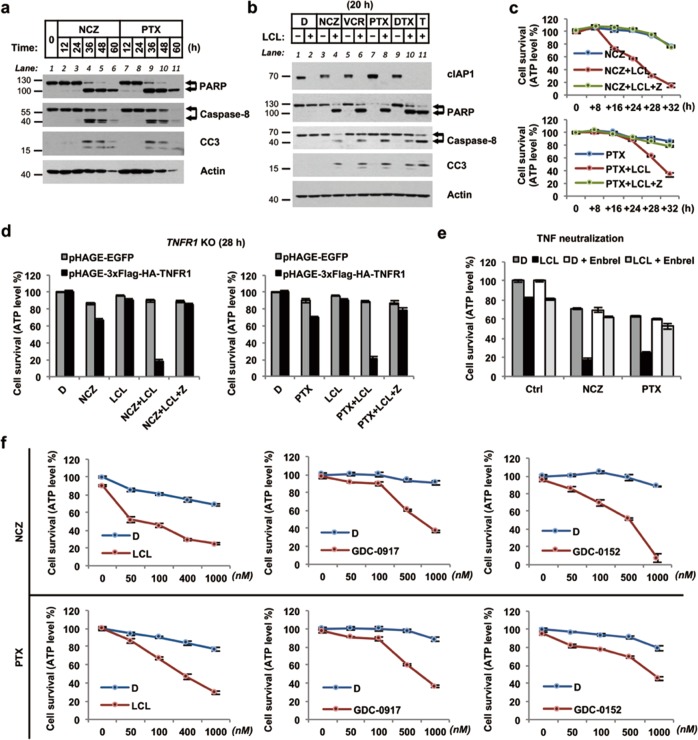
Smac mimetics reduce adverse toxicity of MTAs by potentiating memTNF-mediated apoptosis. **a** Immunoblotting analysis of apoptosis markers using whole cell lysates from MTA-treated HeLa cells. Cells were treated with 1 µM NCZ or PTX for indicated time. **b** Immunoblotting analysis of apoptosis markers using whole cell lysates from MTAs and LCL161 co-treated HeLa cells. Cells were treated with 100 nM MTAs or 20 ng/ml recombinant/soluble TNF (T) in the presence or absence of 100 nM LCL161 as indicated for 20 h. **c** Time course effect of LCL161 on MTA-induced cell death in HeLa cells. **d** Effect of *TNFR1* knockout on MTAs and LCL161 co-treatment induced apoptosis in HeLa cells. Cells were treated as indicated for 28 h. **e** Effect of TNF neutralization on MTAs and LCL161 co-treatment induced cell death in HeLa cells. E, Enbrel. **f** Dose-dependent effect of NCZ (upper) or PTX (lower) treatment on HeLa cells in the presence or absence of 100 nM LCL161 (left), 100 nM GDC-0917 (middle), or 100 nM GDC-0152 (right). D, DMSO; NCZ, nocodazole; PTX, paclitaxel; LCL, LCL161; Z, z-VAD. Cell viability was determined by measuring ATP levels. The data are represented as mean ± SEM of duplicate wells (**a**, **c**–**e**). Results are reported from one representative experiment. Experiments were repeated independently three (**b**–**e**, and LCL161 in **f**) or two (**a**, GDC-0917 and GDC-0152 in **f**) times

In HeLa cells, MTA-alone induced significant apoptosis over 36 h (Fig. 5a). However, we found that, MTAs and LCL161 co-treatment triggered significant caspase-dependent apoptotic cell death within 20 h, much more rapid than MTA-alone-induced apoptosis commence (Fig. 5b, c). Then we tested the role of LCL161 in this combinatory treatment and found LCL161 did not contribute to MAPK activation, c-Jun upregulation, or memTNF deposition as MTAs did (Supplementary Fig. 7). In consistence with this, the MTA and LCL161 co-treatment-induced apoptosis was abolished in *TNFR1* knockout cells; knockdown of *TNF* also blocked MTAs/LCL161-induced apoptosis (Supplementary Fig. 6c); and apoptosis could be restored through *TNFR1* gene re-expression (Fig. 5d). The human TNF receptor fusion protein etanercept (Enbrel^®^), which can neutralize TNF, has been used to treat auto-immune diseases in the clinics [50, 51]. When we used Enbrel to neutralize TNF in HeLa cells, MTAs/LCL161-induced apoptosis was prevented (Fig. 5e). These results show that Smac mimetics sensitize cells to MTAs to trigger more rapid and extensive TNFR1-dependent apoptosis. As expected, upon caspase inhibition with the pan-caspase inhibitor z-VAD, the MTAs/LCL161-induced apoptosis was switched to necroptosis in RIP3-expressing cells (Supplementary Fig. 6d, e). In cell lines that are sensitive to TNF-induced both apoptosis and necroptosis, such as 22Rv1 (a human prostate cancer cell line), NCI-H358 (a human lung cancer cell line), and BxPC-3 (a human pancreatic cell line), MTA-induced apoptosis could be potentiated by Smac mimetic; and z-VAD switched MTA/Smac mimetic-induced apoptosis into necroptosis, which could be further blocked by necroptosis inhibitor Nec-1 (Supplementary Fig. 8). In addition to LCL161, we found that co-treatments comprising MTAs and other Smac mimetics resulted in a similar potentiation of apoptosis in HeLa cells (Fig. 5f).

### Combination therapies comprising MTAs and Smac mimetics elicit robust cell death in a broad spectrum of human carcinoma cell lines

**Fig. 6:**
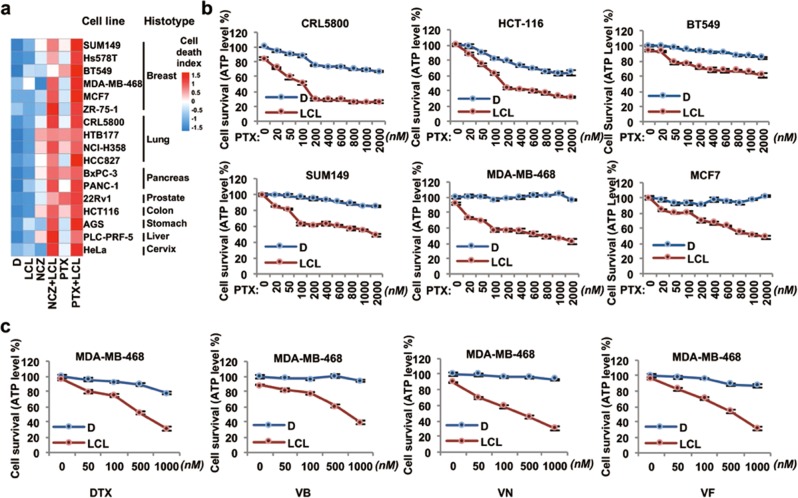
MTAs synergize with Smac mimetics to induce massive apoptosis in various human carcinoma cell lines. **a** A panel of human carcinoma cell lines was tested for MTAs and LCL161 co-treatment induced apoptosis. Heat map analysis of cell death index was calculated based on ATP levels. **b** Dose-dependent effect of NCZ (upper) or PTX (lower) treatment on HCT116 (colon cancer), MDA-MB-468 (breast cancer), and BT549 (breast cancer) cells. **c** Dose-dependent effect of DTX (docetaxel), VB (vinblastine), VN (vinorelbine), or VF (vinflunine) treatment on MDA-MB-468 cells in the presence or absence of 100 nM LCL161. D, DMSO; NCZ, nocodazole; PTX, paclitaxel; LCL, LCL161. Cell viability was determined by measuring ATP levels. The data are represented as mean ± SEM of duplicate wells. Results are reported from one representative experiment. Experiments were repeated independently twice (**a**–**c**)

We then tested the co-treatments of MTAs and Smac mimetics on 17 human cancer cell lines, representing eight different histotypes, and found that all these human cancer cell lines were highly vulnerable to these combination treatments (Fig. 6a). Moreover, even a small dose of the IAP antagonists drastically increased the efficacy of MTAs (Fig. 6b and Supplementary Fig. 6f–i). The sensitization effect of Smac mimetics was broad, since cell death induced by many other MTA drugs (e.g., DTX, vinblastine, vinorelbine, or vinflunine) was also potentiated by them (Fig. 6c).

### MTAs and Smac mimetics synergistically induce memTNF-dependent complete regression of human breast cancers

**Fig. 7:**
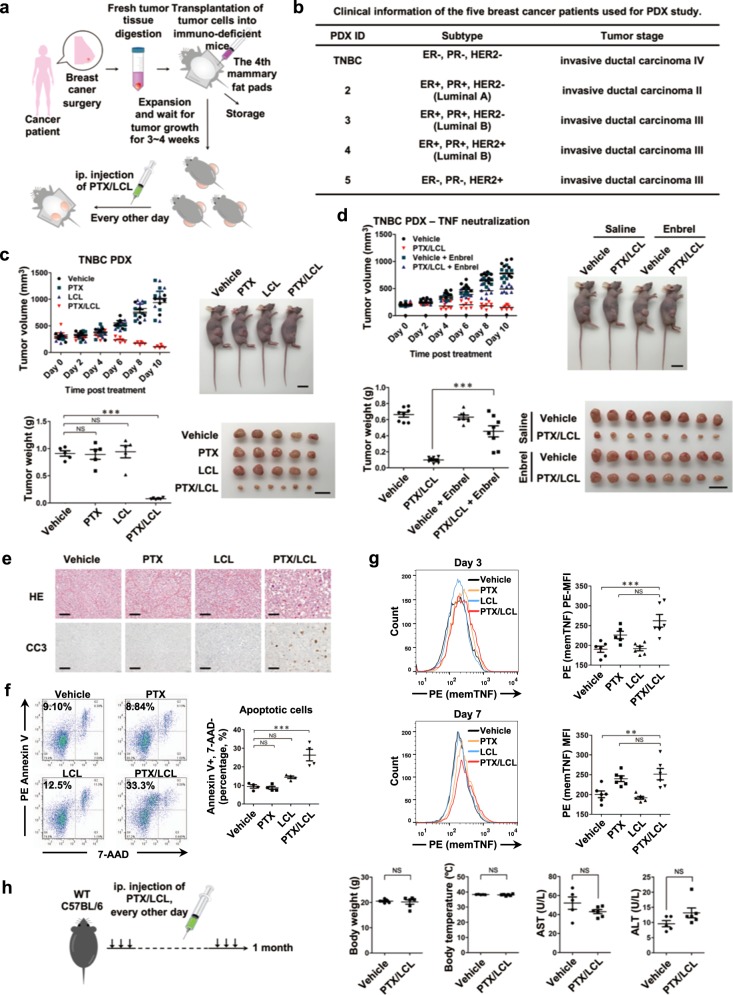
MemTNF-signaling-mediated apoptosis serves as the mechanism of action (MOA) for MTA/S-induced complete regression of human breast cancer. **a** Schematic experimental design for combinatory use of PTX and LCL161 (LCL) in breast cancer PDX, as described in the “Methods” section. **b** Clinical information of breast cancer patients used for PDX study. **c** Effect of PTX and LCL161 combinatory treatment on TNBC PDX. Athymic nude mice bearing 200–300 mm^3^ TNBC PDX were treated with vehicle or with 20 mg/kg PTX and/or 25 mg/kg LCL161. Upper left panel depicts tumor growth, upper right shows representative image of tumor-bearing mice, lower left documented final tumor weight, and lower right represents the image of the final tumors. Vehicle, PTX, and LCL, *n* = 5; PTX/LCL, *n* = 6. Scale bars, 2 cm. **d** Effect of in vivo TNF neutralization on PTX/LCL co-treatment of TNBC PDX. PDX was developed and treated as described in **c**. Enbrel (20 mg/kg) was treated every 3 days. *n* = 8 for each group. Scale bars, 2 cm. **e** 10 days after treatment as in **c**, tumors were isolated for histology (H&E staining) and apoptosis (cleaved caspase-3 IHC) analysis. *n* = 5 for each group. Scale bar, 200 µm. **f** Quantification of apoptotic cells in tumors (right panel), assessed by flow cytometry (left panel). Numbers in quadrants (top left areas; Annexin V^+^ 7-AAD^−^) indicate apoptotic cell percentage in each group. *n* = 4 for each group. **g** Membrane-bound TNF was assayed by flow cytometry after 3-day or 7-day drug treatment. *n* = 5–6 for each group. **h** In vivo toxicity test for PTX/LCL161 combinatory treatment on wild-type C57BL/6 mice. Mice were treated with vehicle or with 20 mg/kg PTX and 25 mg/kg LCL161. Body weight, body temperature, AST, and ALT were all measured at the end of the study. *n* = 6 for each group. PTX, paclitaxel; LCL, LCL161. All graphs show mean ± SEM. *p* values for **c**, **d**, **f**, and **g** were determined by the one-way ANOVA test, followed by Tukey’s multiple comparison post-test; *p* values for body weight and ALT in **h** were determined by Mann–Whitney *U*-test; *p* values for body temperature and AST in **h** were determined by two-tailed unpaired Student’s *t*-test with Welch’s correction. NS not significant; *p < 0.05; **p < 0.01; ***p < 0.001. Results are reported from one representative experiment. Experiments were repeated independently for three (**c)** or two (**d**–**h)** times

To evaluate MTA and Smac mimetic co-treatments in vivo, we used five different subtypes of breast cancer patient-derived xenografts (PDXs; Fig. 7a, b), including triple negative breast cancer (TNBC), a very aggressive and deadly subtype of breast cancer [52]. We chose LCL161 as the Smac mimetic to test the action of co-treatment with PTX [53]. When the xenograft tumors reached certain volume, we began intraperitoneal (i.p.) injection in mice every other day with vehicle or PTX and/or LCL161. Strikingly, the animals in the combination treatment cohort showed complete tumor regression in all subtypes (Fig. 7c and Supplementary Fig. 9a, c, e, and g). In contrast, neither of the two monotherapies had any effect on the breast cancer PDX tumors. We also found that Enbrel drastically reduced the tumor-reducing effects of PTX and LCL161 (Fig. 7d).

Immunohistochemical staining revealed that caspase-3 activation was widespread in tumors co-treated with PTX and LCL161 (Fig. 7e and Supplementary Fig. 9b, d, f, and h). Annexin V and 7-AAD staining also showed more apoptotic cells in the PTX/LCL-treated group (Fig. 7f). Furthermore, flow cytometric analysis showed memTNF upregulation in PTX-treated and PTX/LCL combination-treated tumors (Fig. 7g). These results demonstrate that memTNF signaling mediates the in vivo anti-tumor effects of the MTA/S combination treatment.

Importantly, this combination therapy was well tolerated by the mice. Pharmacodynamics and safety experiments on WT C57BL/6 mice revealed that no liver damage was detected (no elevation in AST or ALT levels). Furthermore, there was no additional body weight loss or body temperature drop in the combination-treated mice (Fig. 7h).

## Discussion

A distinguishing hallmark of cancer cells is that they lose contact inhibition, which results in uncontrolled growth and invasiveness, especially in malignant solid tumors [54]. Our study found that MTAs utilize the interacting tumor cells to induce cancer-cell-to-cancer-cell killing, which shrinks the entire tumor, regardless of their proliferative status. For this reason, the dose threshold requirement is much lower than the level required for direct cell killing by drugs such as mitosis-specific agents. Owing to the severe systemic inflammatory response syndrome (SIRS) that they apparently invariably evoke, systemic therapies based on solTNF are totally unsuitable for use in the clinic, even when only subtherapeutic doses of solTNF are administered [55, 56, 57]. A previous study on genetically engineered mice without secreted TNF but with regulated memTNF expression has shown that memTNF exhibits suboptimal pro-inflammatory function in vivo and is inferior to secreted TNF in driving autoimmune diseases [58, 59]. Therefore, activating memTNF can potentially ensure strictly localized action without harmful systemic effects.

Our study establishes that the extent of memTNF accumulation and the subsequent cancer-cell-to-cancer-cell killing is highly restricted to MTA treatment and does not occur under treatments with other non-MTA cell-cycle-arresting agents. Our work elucidates that MTAs have crucial roles in killing both mitotic and non-mitotic cells in PDXs; therefore, they are effective against even some very slowly growing tumors. Meanwhile, our results suggest that the clinical disappointment regarding mitosis-specific inhibitors may at least partly be ascribed to their inability to promote cytotoxic memTNF expression in a mass of living tumor.

JNK belongs to the mitogen-activated protein kinase family and is responsive to multiple cellular stress stimuli [60, 61], and our work clearly shows that microtubule disturbance must be understood as a previously unappreciated stress stimulus that results in JNK/c-Jun activation. Continued studies on the exact mechanistic regulation through which microtubule disturbance leads to JNK/c-Jun activation should be fruitful for many years to come.

It has been known for more than 20 years that high doses of Taxol promotes the secretion of solTNF from macrophages, which occur independently of its microtubule-binding effects [62–65]. Specifically, the dose for PTX to stimulate macrophage activation (e.g., 10, 30, and 100 μM as reported) was much higher than we reported here (≤1 μM). Given that MTAs can elicit cancer cell death via their stimulation of solTNF release from macrophages and the increase of memTNF in cancer cells, the in vivo cell-killing contributions of both mechanisms must now be considered.

The reported preclinical studies and clinical trials reported the observation that high anticancer efficacy of PTX/LCL161 co-therapies (including breast and lung cancers) [47–49]. Our discovery of the essential role of memTNF-mediated cell death in these effects defines the mechanistic rationale that can explain how this observed synergism actually occurs. And the generality of such mechanisms is highlighted by our results showing the same efficacy for multiple cancer cell lines representing different histotypes using different versions of Smac mimetics. Our work thus opens up a new range of prospects for developing novel co-therapies against many solid tumor types.

The therapeutic efficacy of MTA drugs is often accompanied with undesirable side effects, such as peripheral and autonomic neuropathy [66, 67]. Our work strongly advocates for exploration of the idea that memTNF could be useful as a highly informative and perhaps specific biomarker of patient responsiveness to MTAs.

## Methods

### Plasmids

For stable expression in L929 cells, EGFP (non-tagged), mouse RIP1, RIP3, MLKL, TNFR1, TNF (all Flag-tagged), and human TNFR1 (3 × Flag-HA-tagged) were cloned into pHAGE plasmid, which was kindly provided by W. Zou (SIBCB, China). Point mutations were introduced by site-directed PCR strategy. For CRISPR-Cas9-mediated gene knockout, specific sgRNAs were cloned into pX330-mCherry plasmid, which was kindly provided by J. Li (SIBCB, China).

### Antibodies and reagents

For immunoblotting, anti-MLKL (mouse specific, Biorbyt, #orb32399; human specific, Abcam, #ab184718), anti-phospho-MLKL (mouse specific, Abcam, #ab196436; human specific, Abcam, #ab187097), anti-TACE (Abcam, #ab39163), anti-RIP1 (Cell Signaling, #3493), anti-COX IV (Cell signaling, #11967S), anti-cJun (Cell signaling, #9165S), anti-phospho-cJun (Ser73, Cell signaling, #9164S), anti-JNK (Cell signaling, #9258), anti-phospho-JNK (Thr183/Tyr185, Cell signaling, #4668S), anti-p38 (Cell signaling, #9212S), anti-phospho-p38 (Thr180/Tyr182, Cell signaling, #9215S), anti-Erk2 (Santa cruz, #sc-154), anti-phospho-p44/42 MAPK (Erk1/2) (Thr202/Tyr204, Cell signaling, #4370S), anti-MEK1 (Santa cruz, #sc-219), anti-phospho-MEK1/2 (Ser217/221, Cell signaling, #9154S), anti-IκBα (Santa cruz, #sc-371), anti-RelB (Cell signaling, #4922T), anti-PARP (Cell signaling, #9542L), anti-capsase8 (Cell signaling, #9746), anti-cleaved caspase 3 (Asp175, Cell Signaling, #9661), anti-human TNFR1 (Cell signaling, #3736), anti-mouse RIP3 (Prosci, #2283), anti-mouse TNF (R&D, #AF-410-SP), anti-mouse TNFR1(R&D, #BAF425), anti-p65 (RelA, Santa cruz, #sc-8008), anti-actin (Sigma-Aldrich, #029K4838), and anti- Flag-HRP (Sigma-Aldrich, #A8592) antibodies were used. For immunohistochemistry (IHC), anti-cleaved caspase 3 (Asp175, Cell Signaling, #9661) antibody was used. For TNF neutralization, anti-mouse TNF (15 μg/ml, R&D, #AF-410-SP) antibody and Enbrel (20 μg/ml for in vitro cell culture; 20 mg/kg for in vivo treatment of nude mice, Pfizer) were used. For flow cytometry analysis, PE Annexin V apoptosis detection kit (BD Pharmingen, #559763), PerCP-Cy5.5 rat anti-mouse TNF (clone MP6-XT22, BD Pharmingen, #560659), PE mouse anti-human TNF (clone MAb11, BD Pharmingen, #559321), and PE-Cy7 rat anti-mouse CD45 (clone 30-F11, BD Pharmingen, #561868) were used. Recombinant TNF was purified in our lab. Nocodazole (#S2775), PTX (#S1150), TAPI-1 (#S7434), BB-94 (#S7155), BB-2516 (#S7156), and SP600125 (#S1460) were purchased from Selleck. LCL161 (#HY-15518), GDC-0917 (#HY-15835), GDC-0152 (#HY-13638), JNK-IN-8 (#HY-13319), and CC-930 (#HY-15495) were purchased from MedChemExpress. Nec-1 (#N9037) was purchased from Sigma. Nec-1s (#2263) was purchased from BioVision. ActD (#A0977) was purchased from LKT Laboratories. All other inhibitors were obtained from National Compound Resource Center, unless otherwise noted.

### Cell culture

L929, HeLa, Hs578T, MDA-MB-468, MCF7, CRL5800, HTB177, BxPC-3, PANC-1, 22Rv1, HCT116, AGS, and PLC-PRF-5 cells were cultured in Dulbecco’s modified Eagle’s medium (DMEM) supplemented with 10% fetal bovine serum (FBS) and 100 units/ml penicillin/streptomycin. BT549, ZR-75-1, NCI-H358, and HCC827 cells were maintained in RPMI-1640 medium supplemented with 10% FBS and 100 units/ml penicillin/streptomycin. SUM149 cells were cultured in Ham’s F-12 medium supplemented with 5% FBS, 5 μg/ml insulin, 1 μg/ml hydrocortisone, and 4 μg/ml gentamycin. All cells were cultured at 37 °C in a 5% CO_2_ incubator. All cell lines were tested to be mycoplasma-negative by the standard PCR method.

### Cell death assay

L929 cells were seeded into 96-well plate (5000/well) and allowed to grow for 24 h, and then treated with 500 nM NCZ, 500 nM VCR, 1 µM PTX, or 500 nM DTX in the presence or absence of 10 µM Nec-1 for another 24 h. The intracellular ATP levels of the remaining cells were measured to determine cell viability. For human carcinoma cell lines, 5000–8000 cells/well were seeded into 96-well plate and allowed to grow for 24 h. The cells are then treated with 100 nM NCZ, 100 nM VCR, 100 nM PTX, or 100 nM DTX in the presence or absence of 100 nM Smac mimetics (LCL161, GDC-0917, and GDC-0152) and/or 20 µM z-VAD for 24 h. The intracellular ATP levels of the remaining cells were measured to determine cell viability. Cell viabilities (the intracellular ATP levels of the remaining cells) were measured with the CellTiter-Glo Luminescent Cell Viability Assay Kit (Promega) according to the manufacturer’s instructions.

### SYTOX Green staining and membrane leakage assay

SYTOX Green (Invitrogen) was added to the cell culture medium at a final concentration of 100 nM to trace the plasma membrane breakdown 10 min before microscopic imaging. For membrane leakage assay, SYTOX Green fluorescence intensity was measured by a BioTek microplate reader using the wavelength of 485/520 nm, named as induced fluorescence. The cells were then lysed by 0.1% Triton X-100, and the SYTOX Green fluorescence intensity was measured as maximal fluorescence. Membrane leakage was calculated as (induced fluorescence–background fluorescence)/(maximal fluorescence–background fluorescence) × 100%.

### Triton X-114 phase-separation assay

Triton X-114 phase-separation assay was performed as described elsewhere [15]. Briefly, L929 cells were collected, washed twice with PBS and re-suspended in Triton X-114 lysis buffer. After incubation on ice for 30 min, the samples were centrifuged at 15,000 × *g* at 4 °C for 10 min. The supernatant was harvest and warmed at 30 °C for 3 min. After centrifugation at 1500 × *g* for 5 min at RT, the upper aqueous layer and the lower detergent enriched layers were collected, respectively. Detergent-enriched layer was washed with basal buffer and diluted to the same volume as aqueous fraction.

### Triton X-100 fractionation

L929 cells were collected, washed twice with PBS and re-suspended in Triton X-100 lysis buffer. After incubation on ice for 20 min, the samples were centrifuged at 12,700 rpm at 4 °C for 10 min. The Triton X-100 soluble supernatant was saved as S fraction, while the Triton X-100 insoluble pellet was washed with PBS and saved as P fraction.

### CRISPR-Cas9-mediated gene knockout and lenti-virus infection

sgRNAs were cloned into pX330-mCherry. L929 or HeLa cells were co-transfected with two sgRNA-expressing plasmids by PolyJet transfection reagent according to the manufacturer’s instructions (SignaGen Laboratories). 24 h after transfection, the cells were digested with trypsin and resuspended with DMEM. mCherry-positive live cells were sorted by the BD FACS Aria flow cytometer and seeded in 10-cm Petri dishes (200 cells/dish). After 7 days, single cell clones were picked up by trypsin digestion. qRT-PCR and immunoblotting were used to screen for the KO clones. sgRNAs sequences are as follows: mouse *Rip3* (GAGGGTTCGGAGTCGTGTTC, ACCCTCCCTGAAACGTGGAC), mouse *Mlkl* (GCACACGGTTTCCTAGACGC, CGCTAATTTGCAACTGCATC), mouse *Tnfr1* (TGTCACGGTGCCGTTGAAGC, GTGAGTGACTGTCCGAGCCC), mouse *Tnf* (AGAAAGCATGATCCGCGACG, TCGGGGTGATCGGTCCCCAA), mouse *Jun* (GGTCCGAGTTCTTGGCGCGG, GCTGACCGGCTGTGCCGCGG), and human *TNFR1* (ATATACCCCTCAGGGGTTAT, GTGGTTTTCTGAAGCGGTGA).

For stable rescued cell lines, L929 or HeLa KO cells were seeded in 6-well plate and infected with indicated pHAGE virus in the presence of 10 µg/ml polybrene. 48 h after infection, cells were selected with 10 µg/ml puromycin. Gene and protein re-expression were confirmed by qRT-PCR and immunoblotting, respectively.

Apart from the KO/rescued clones shown in the manuscript, the data are further confirmed by another L929 *Rip1* KO clone, three other L929 *Rip3* KO clones, two other L929 *Mlkl* KO clones, two other L929 *Tnfr1* KO clones, four other L929 *Tnf* KO clones, and two other L929 *Jun* KO clones.

### ELISA analysis of soluble TNF

WT L929, *Tnf* KO L929, and Raw264.7 cells were grown in 12-well plate. WT and *Tnf* KO L929 cells were stimulated with 500 nM NCZ or 1 µM PTX in 300 µl fresh DMEM medium for 18 h; Raw264.7 cells were stimulated with 100 ng/ml LPS in 300 µl fresh DMEM medium for 3 h. Then, culture medium was collected and centrifuged at 8000 rpm for 2 min to remove cell debris. 50 µl aliquots were used to measure TNF concentration with mouse TNF ELISA Kit (eBioscience). Two independent wells were performed.

### CM feeding assay

Raw264.7 cells and L929 cells were first seeded in 6-well plate. Raw264.7 cells were treated with 100 ng/ml LPS in the presence or absence of 10 µM TACE inhibitors for 3 h. L929 cells were treated with 500 nM NCZ for indicated time. CM was collected and filtered by passing through a 0.22 µm filter. The target naïve L929 cell were seeded in 96-well plate. For Raw264.7-CM feeding, 100 µl Raw264.7 CM were added to each well of naïve L929 cells. Cell viability was measured 9 h later. For L929-CM feeding, cell viability was measured 12 h later.

### Crude plasma membrane (P100) isolation

L929 cells were collected, washed twice with PBS and re-suspended in sucrose-containing buffer (10 mM Tris–HCl, pH 7.4, 250 mM sucrose, 5 mM MgCl_2_). After incubation on ice for 30 min, the cells were homogenized with ultrasonication. Cell homogenate was firstly centrifuged at 16,000 × *g* for 10 min to remove nuclei and cellular debris. The supernatant was then collected and re-centrifuged at 100,000 × *g* at 4 °C for 1 h (HITACHI CS150GX). The pellet was collected as crude membrane fraction (P100) and the supernatant was collected as cytosolic fraction (S100).

### TNF neutralization

L929 cells were seeded in 96-well plate. 15 µg/ml TNF-neutralizing antibody (α-TNF) was added 2 h before NCZ or PTX treatment. Cell viability was measured by ATP levels.

### Operetta imaging and IncuCyte analysis

For Operetta imaging, L929 *Tnfr1* KO cells stably expressing EGFP and L929 *Tnf* KO cells were seeded in 96-well plate at the ratio of 5:1, then treated with 500 nM NCZ for 24 h, and stained with PI for 10 min. Images were taken by operetta (PerkinElmer) using the Harmony software. EGFP was imaged at 460–490/500–550 nm and PI was imaged at 520–550/560–630 nm. For IncuCyte dead cell analysis, L929 *Tnf* KO and L929 *Tnfr1* KO cells were seeded in 96-well plated as indicated. Cells were treated with DMSO or 500 nM NCZ for 24 h and stained with 100 nM SYTOX Green. Cells were imaged (450–490 nm/500–530 nm) and quantified by IncuCyte FLR.

### Quantitative real-time PCR (qRT-PCR)

Total RNA was extracted by Trizol (Life technology) and reverse transcription was performed with M-MLV transcriptase (Promega). Quantitative PCR was performed with gene specific primers (5′–3′): mouse *Gapdh* (forward, AGGTCGGTGTGAACGGATTTG; reverse, GGGGTCGTTGATGGCAACA); mouse *Rip1* (forward, GAAGACAGACCTAGACAGCGG; reverse, CCAGTAGCTTCACCACTCGAC); mouse *Rip3* (forward, TGTCAAGTTATGGCCTACTGGTGCG; reverse, AACCATAGCCTTCACCTCCCAGGAT); mouse *Mlkl* (forward, AATTGTACTCTGGGAAATTGCCA; reverse, TCTCCAAGATTCCGTCCACAG); mouse *Tnfr1* (forward, CCGGGAGAAGAGGGATAGCTT; reverse, TCGGACAGTCACTCACCAAGT); mouse *Tnf* (forward, CAGGCGGTGCCTATGTCTC; reverse, CGATCACCCCGAAGTTCAGTAG); mouse *Jun* (forward, CCTTCTACGACGATGCCCTC; reverse, GGTTCAAGGTCATGCTCTGTTT); mouse *Rela* (forward, AGGCTTCTGGGCCTTATGTG; reverse, TGCTTCTCTCGCCAGGAATAC); mouse *Relb* (forward, CCGTACCTGGTCATCACAGAG; reverse, CAGTCTCGAAGCTCGATGGC); human *GAPDH* (forward, CCAGAACATCATCCCTGCCT; reverse, CCTGCTTCACCACCTTCTTG); human *TNFR1* (forward, AACGAGTGTGTCTCCTGTAGT; reverse, GGAGTAGAGCTTGGACTTCCAC); human *TNF* (forward, GAGGCCAAGCCCTGGTATG; reverse, CGGGCCGATTGATCTCAGC); human *JUN* (forward, AACAGGTGGCACAGCTTAAAC; reverse, CAACTGCTGCGTTAGCATGAG). The mRNA level of target genes was normalized to that of GAPDH.

### RNA sequencing and data processing

L929 cells were treated with 500 nM NCZ or 1 µM PTX for indicated time. Total RNA was extracted with Trizol (Life technology) and quantity control was performed on an Agilent Bioanalyzer 2100 (Agilent Technologies). Qualified total RNA was further purified by RNeasy micro kit (Cat#74004, QIAGEN, GmBH, Germany) and RNase-Free DNase Set (Cat#79254, QIAGEN, GmBH, Germany). cDNA libraries were constructed with an TruSeq-stranded mRNA kit (Illumina) using Poly-A selection. Samples were sequenced on illumina HiSeq^@^ 2500 system by paired-end.

Clean reads were mapped to mm10 genome using Tophat (version 2.1.1). Gene expression level was calculated by Cufflinks (version 2.2.1) based on the mm10 refFlat annotation database from the UCSC genome browser.

### siRNA knock down

Cells were seeded in 96-well plate or 6-well plate and siRNA oligos were transfected with Lipofectamine RNAiMAX reagent following manufacturer’s instructions (Invitrogen). All genes were targeted by four specific siRNA oligos from Dharmacon: mouse *Rela* (GGAGUACCCUGAAGCUAUA, GAAGAAGAGUCCUUUCAAU, UAUGAGACCUUCAAGAGUA, GAAUCCAGACCAACAAUAA), mouse *Relb* (UGGAAAUCAUCGACGAAUA, GCUACGGUGUGGACAAGAA, GAAGAUCCAGCUGGGAAUU, GAAGAUCCAGCUGGGAAUU), human *TNF* (GCCCGACUAUCUCGACUUU, GCGUGGAGCUGAGAGAUAA, UGACAAGCCUGUAGCCCAU, CCAGGGACCUCUCUCUAAU).

### In vitro TACE enzymatic assay

L929 cells were treated with 500 nM NCZ or 1 µM PTX for indicated time. Cell lysate and membrane fractions were prepared and mixed with the fluorogenic TACE substrate peptide. Fluorescence intensity at Ex/Em = 490 nm/520 nm was measured in a BioTek microplate reader according to manufactures instructions (ANASPEC SensoLyte 520 TACE Activity Assay Kit, 72085).

### Animals

For in vivo tumorigenicity of L929 fibrosarcoma and PDXs, 6-week-old female athymic nude mice were used. For in vivo toxicity assays of PTX/LCL161, 8-week-old female WT C57BL/6 mice were used. All mice were obtained from Shanghai SLAC Laboratory Animal Center of Chinese Academy of Sciences (Shanghai, China) and housed in standard IVC cages under specific pathogen-free conditions in a 12-h light/dark cycle (light between 07:00 and 19:00) in a temperature-controlled room (22 ± 1 °C) at Institute of Biochemistry and Cell Biology. All animal experiments were approved by the Institutional Animal Care and Use Committee (IACUC) of Shanghai Institute of Biochemistry and Cell Biology, Chinese Academy of Sciences and complied with all relevant ethical regulations. Tumor sizes were within the limit permitted by SIBCB IACUC.

### L929 fibrosarcoma model

L929 fibrosarcoma cells (1 × 10^6^) were subcutaneously injected into the dorsal part of athymic nude mice. Tumor size was measured every 2 days and tumor volume was calculated as *a* × *b*^2^ × 0.5 (*a*, maximum diameter; *b*, minimum diameter). When tumor volume reached ~300 mm^3^, the tumor-bearing mice were randomly assigned into three groups and received vehicle, 5 mg/kg VCR, or 5 mg/kg Nec-1s in combination with VCR through intraperitoneal injection every other day, until the most effective group reached complete regression. At the end of the study, the mice were sacrificed for tumor dissection.

### PDX model

Fresh tumor tissues were isolated from stable PDX-bearing mice and digested into single tumor cells with collagenase/hyaluronidase (STEMCELL Technologies, #07912). 2 × 10^5^ single tumor cells were mixed with matrigel (BD Biosciences, 354234) and transplanted into the fourth mammary fat pad of athymic nude mice. For TNBC PDX, when the tumor volume reaches 200–300 mm^3^ (about 4 weeks post transplantation), tumor-bearing mice were randomly assigned into four experimental groups (Vehicle, PTX, LCL, and PTX/LCL). Mice were treated with 20 mg/kg PTX or 25 mg/kg LCL161 or both through intraperitoneal injection every other day, for 10 days until the most effective group reached complete tumor regression. PTX and LCL161 were dissolved in DMSO for storage and further diluted with 20% DMSO and 5% Tween-80 for in vivo treatment. Tumor size was measured every 2 days for the growth curve. At the end of the study, the mice were sacrificed with image taken and tumor weights recorded. Tumors were harvest for FACS analysis or histological analysis. For others PDXs, the tumor-bearing mice were randomly assigned into four groups and received the same treatment just as the TNBC PDX when the tumor volume reaches ~800 mm^3^. For in vivo TNF neutralization, Enbrel (Pfizer) was dissolved in sterile 0.9% saline and intraperitoneally injected into mice. The first injection was given 2 h before PTX/LCL treatment and then every 3 days.

### FACS analysis of apoptotic cells and memTNF

Tumors were isolated freshly, cut into small pieces and digested into single tumor cells with collagenase/hyaluronidase. For apoptotic cell analysis, single tumor cells after 3 days of drug treatment were stained with PE Annexin V and 7-AAD according to the manufacturer’s instructions. For memTNF analysis, single tumor cells after 3 or 7 days of drug treatment were stained with PE mouse anti-human TNF antibody and PE-Cy7 rat anti-mouse CD45 antibody at 4 °C for 40 min in the dark. The stained cells were then analyzed on BD Celesta or BD LSRII SORP flow cytometer and data was processed by FlowJo software. Apoptotic cells (Annexin V^+^ 7-AAD^−^) were quantified based on FACS results. CD45-negative live tumor cells were gated for memTNF analysis and mean fluorescence intensity (MFI) of PE (right panel) were shown.

### Histology and IHC

Freshly isolated tumor tissues were fixed overnight in 4% PFA, dehydrated with a graded ethanol series and xylene, and embedded in paraffin. 5 μm paraffin sections were prepared and then stained with hematoxylin and eosin (HE staining) for histological analysis. For IHC, paraffin sections were first deparaffinized and rehydrated. Endogenous hydrogen peroxide was removed by 3% H_2_O_2_ and antigen retrieval was performed in 10 mM citric acid buffer (pH 6.0). The sections were then blocked with 5% normal goat serum and incubated with cleaved caspase 3 (CC3) antibody at 4 °C, overnight. The next day, CC3 antibody was removed. The sections were washed with PBS and incubated with rabbit-HRP for 1 h at RT. After the incubation, the sections were washed with PBS and then developed with ImmPACT DAB peroxidase substrate kit (Vector, SK-4105). The sections were then counterstained with hematoxylin, dehydrated with ethanol and xylene, and mounted with mounting medium. Images were acquired on Olympus BX53 microscope.

### In vivo drug toxicity test

WT C56BL/6 mice were given intraperitoneal injection of 20 mg/kg PTX and 25 mg/kg LCL161 every other day for one month. At the end of the study, body weight and body temperature were recorded. For evaluation of alanine aminotransferase (ALT) and aspartate aminotransferase (AST) activity, fresh blood was centrifuged at 3000 × *g* for 10 min and the supernatant plasma was used.

### Quantification and statistical analysis

Statistical analyses were performed with GraphPad Prism 5 (GraphPad Software, Inc.). Statistical significance was determined as indicated in the figure legends. NS, not significant; *p < 0.05, **p < 0.01, ***p < 0.001. The data distribution was first checked using a Kolmogorov–Smirnov test. For comparison between two groups, if the data fitted a normal distribution, a two-tailed unpaired Student’s *t*-test was used when variances were similar whereas a two-tailed unpaired Student’s *t*-test with Welch’s correction was used when variances were different; if the data did not fit a normal distribution, a Mann–Whitney *U*-test was used. For multiple comparisons, if the data fitted a normal distribution, a one-way ANOVA test was used, followed by Dunnett’s post-test when comparing each group to the Vehicle group, or followed by Tukey’s post-test when comparing all pairs of groups; if the data did not fit a normal distribution, a Kruskal–Wallis test was used, followed by Dunns’s post-test. For comparison of the effect of different treatment on tumor growth, a two-way ANOVA was used. Data are mean ± SEM.

## Supplementary information

supplementary figure legend

Supplementary Fig. 1

Supplementary Fig. 2

Supplementary Fig. 3

Supplementary Fig. 4

Supplementary Fig. 5

Supplementary Fig. 6

Supplementary Fig. 7

Supplementary Fig. 8

Supplementary Fig. 9

attribution of authorship

DATA SET 1
