# Supplementary material for: Membrane-bound TNF mediates microtubule-targeting chemotherapeutics-induced cancer cytolysis via juxtacrine inter-cancer-cell death signaling
Source: Cell Death Differ. 2019 Oct 23;27(5):1569–87. doi: 10.1038/s41418-019-0441-3 (PMC7206059; doi:10.1038/s41418-019-0441-3)
Supplement: Supplementary file 1 — supplementary figure legend [file 41418_2019_441_MOESM1_ESM.docx]

**Supplementary Fig. 1 | MTA-induced necroptosis is RIP1-dependent in L929 cells.**

**a**-**d**, Effect of RIP1 kinase activity on MTA-induced necroptosis in L929 cells. Wild-type or kinase-dead form RIP1 (K45A) were stably expressed in *Rip1* KO L929 cells by pHAGE infection. RIP1 re-expression was detected by qRT-PCR (**a**) and immunoblotting (**b**). Recombinant TNF induced cell death (**c**) was used as positive control for MTA-induced TNF-mediated necroptosis (**d**) in these cell lines. **e**, Time course of NCZ-induced necroptotic cytolysis on L929 cells. **f**, Dose-dependent necroptotic cytolysis effect of NCZ on L929 cells. Membrane leakage was determined by measuring SYTOX Green fluorescence intensity at 485/520 nm.

D, DMSO; NCZ, nocodazole; VCR, vincristine; PTX, paclitaxel; DTX, docetaxel. Cell viability was determined by measuring ATP levels. The data are represented as mean ± SEM of duplicate wells (**a**, **c-f**). Results are reported from one representative experiment. Experiments were repeated independently for four (**e**), three (**a**-**d**), or two (**f**) times.

**Supplementary Fig. 2 | The necroptotic cytotoxicity profile of cell-cycle-arresting agents on L929 cells.**

A panel of 35 cell-cycle-arresting agents was tested for necroptosis effect on L929 cells. Heat map analysis of cell death index was calculated based on ATP levels*.* The data are represented as mean ± SEM of duplicate wells. Results are reported from one representative experiment. Experiments were repeated twice independently.

**Supplementary Fig. 3 | MTA-induced cell death is RIP3- and MLKL-dependent in L929 cells**.

**a**, Immunoblotting analysis of MLKL phosphorylation in Triton X-100 soluble fraction (S) and the pellet (P) of NCZ- or PTX-treated L929 cells. T, TNF treatment. **b**, **c**, Immunoblotting analysis of time-(**b**) and dose-(**c**) dependent MLKL phosphorylation in whole cell lysates of NCZ-treated L929 cells. **d**, Immunoblotting analysis of MLKL phosphorylation in whole cell lysates of VCR-, PTX-, and DTX-treated L929 cells. T, TNF treatment. **e**-**h**, Effect of *Mlkl* knockout on MTA-induced necroptosis in L929 cells. **i**-**k**, Effect of *Rip3* knockout on TNF-induced necroptosis in L929 cells. MLKL and RIP3 re-expression was detected by qRT-PCR (**e**, **i**) and immunoblotting (**f**, **j**). Recombinant TNF induced cell death (**g**, **k**) were used as positive controls for MTA-induced cell death (**h**) in these cell lines.

D, DMSO; NCZ, nocodazole; VCR, vincristine; PTX, paclitaxel; DTX, docetaxel. Cell viability was determined by measuring ATP levels. The data are represented as mean ± SEM of duplicate wells (**g**, **h**, and **k**). Results are reported from one representative experiment. Experiments were repeated independently for three (**a**-**c**, and **e**-**k**) or two (**d**) times.

**Supplementary Fig. 4 | MTA-induced necroptosis is memTNF-dependent in L929 cells.**

**a**-**c**, Effect of *Tnfr1* knockout on TNF-induced necroptosis in L929 cells. TNFR1 re-expression was detected by qRT-PCR (**a**) and immunoblotting (**b**). Recombinant TNF induced cell death (**c**) was used as positive control for TNF-signaling-mediated necroptosis in these L929 cell lines. **d**, Effect of *Tnf* knockout on recombinant TNF (T)- or z-VAD (Z)-induced necroptosis in L929 cells. **e**, LPS-primed Raw264.7 conditioned medium (CM) was applied to naïve L929 cells. Left panel, a schematic representation of the experimental design. Middle and right panels, cell viability of CM-fed L929 was determined by ATP levels at 9 hours post treatment. **f**, ELISA analysis of solTNF in culture medium of MTA-treated L929 cells (left panel) or LPS-primed Raw264.7 cells (right panel) in the presence or absence of TACE inhibitor TAPI-1. **g**, Overview of crude membrane (P100) fractionation by centrifugation. **h**-**j**, Immunoblotting analysis of membrane-bound TNF in crude membrane factions (P100) of PTX- (**h**), tozasertib- (**i**, right), monastrol- (**j**, left), and RO-3306- (**j**, middle) treated L929 cells. NCZ-treated *Tnfr1* KO (**i**, left) and WT (**j**, right) L929 cells were used as positive controls for TNF in P100 fractions. **k**, Cell density-dependent necroptotic effect of MTA-induced cell death in L929. Cells were seeded into 96-well plate at the indicated density and treated with 500 nM NCZ or 1 µM PTX for 24 hours.

D, DMSO; NCZ, nocodazole; PTX, paclitaxel; Z, z-VAD. Cell viability was determined by measuring ATP levels. The data are represented as mean ± SEM of duplicate wells (**c-f, k**). Results are reported from one representative experiment. Experiments were independently repeated four (**d**), three (**a**-**c** and **h**), or two (**e, f** and **i**-**k**) times.

**Supplementary Fig. 5 Transcriptome profiling by RNA-sequencing of MTA-treated L929 cells.**

**a**, **b**, Cluster analysis of transcription factors (based on FPKM of RNA-sequencing) of NCZ- (**a**) or PTX (**b**)- treated L929 cells. Gene list and expression are provided in DATA SET 1. **c**, **d**, Influence of siRNA mediated knock down of *Rela* (**c**) or *Relb* (**d**) on MTA-treated L929 cells. siRNA knock down efficiency was determined by immunoblotting. **e**, **f**, qRT-PCR analysis of *Tnf* mRNA level in NCZ-treated *Rela* (**e**) or *Relb* (**f**) knock down L929 cells. **g**-**i**, qRT-PCR analysis of *Jun* (left) and *Tnf* (right) mRNA level in tozasertib- (**g**), monastrol- (**h**), and RO-3306- (**i**) treated L929 cells. **j**-**l**, Immunoblotting analysis of JNK and c-Jun activation in whole cell lysates of tozasertib- (**j**), monastrol- (**k**), and RO-3306- (**l**) treated L929 cell. **m**, RNA-sequencing analysis of *Tace* and *Rhbdf2* gene expression patterns during MTAs treatment. UCSC genome browser images depict calculated FPKM (fragments per kilobase of transcript per million mapped reads) values in RNA-sequencing data.

D, DMSO; NCZ, nocodazole; PTX, paclitaxel. Cell viability was determined by measuring ATP levels. The data are represented as mean ± SEM of duplicate wells (**c**, **d**). Results are reported from one representative experiment. Experiments were repeated independently for three (**c**, **d**) or two (**e**, **f**, and **g**-**l**) times.

**Supplementary Fig. 6 | MTAs synergize with Smac mimetics to induce apoptosis in a variety of human carcinoma cell lines.**

**a**, **b**, qRT-PCR (**a**) and immunoblotting (**b**) analysis of TNFR1 re-expression in *TNFR1* knockout HeLa cells. **c**, Influence of siRNA mediated knock down of *TNF* on MTAs and LCL161 co-treatment induced apoptosis in HeLa cells. siRNA knock down efficiency was determined by qRT-PCR. **d**, **e**, MTAs induce necroptosis in RIP3-expressing cells. HeLa-RIP3 cells were treated with NCZ (left) or PTX (right) combining LCL161 in the presence or absence of z-VAD for the indicated time (**d**). Cell viability was determined by measuring ATP levels. Immunoblotting analysis of MLKL phosphorylation in MTAs combine LCL161 and z-VAD treated HeLa-RIP3 cells (**e**). **f**-**i**, Dose-dependent effect of NCZ (upper) or PTX (lower) treatment on CRL5800 (**f**), HCT116 (**g**), MDA-MB-468 (**h**), and BT549 (**i**) cells in the presence or absence of 100 nM GDC-0917 (left) or 100 nM GDC-0152 (right).

D, DMSO; NCZ, nocodazole; PTX, paclitaxel; LCL, LCL161; Z, z-VAD. Cell viability was determined by measuring ATP levels. The data are represented as mean ± SEM of duplicate wells (**c**, **d**, and **f-i**). Results are reported from one representative experiment. Experiments were repeated independently for three (**a**-**e**) or two (**f**-**i**) times.

**Supplementary Fig. 7 | Smac mimetic dose not contribute to MAPK activation or memTNF upregulation.**

**a**, **b**, Immunoblotting analysis of cIAP, MAPK pathway (JNK, p38, Erk, MEK, and cJun), and NF-κB pathway (IKBα) in whole cell lysates of NCZ, LCL161 (LCL), or NCZ combines LCL161 (NCZ/LCL)-treated HeLa (**a**) or HCT116 (**b**) cells. **c**, **d**, Flow cytometric analysis of memTNF (PE-conjugated) in NCZ, LCL161 (LCL), or NCZ combines LCL161 (NCZ/LCL)-treated HeLa (**c**) or HCT116 (**d**) cells.

D, DMSO; NCZ, nocodazole; LCL, LCL161. Results are reported from one representative experiment. Experiments were repeated independently for two times.

**Supplementary Fig. 8 | MTAs induce both apoptosis and necroptosis in human cell lines.**

**a**, **d**, **g**, TNF-induced cell death in human prostate cancer cell line 22Rv1 (**a**), human lung cancer cell line NCI-H358 (**d**), and human pancreatic cancer cell line BxPC-3 (**g**). Cells were treated with 40 ng/ml recombinant TNF in the presence or absence of 100 nM LCL161 (LCL), 20 μM z-VAD (Z), or 10 μM Nec-1 as indicated for 24 hours. **b**, **e**, **h**, MTA-induced cell death in 22Rv1 (**b**), NCI-H358 (**e**), and BxPC-3 (**h**) cells. Cells were treated with 100 nM NCZ or PTX in the presence or absence of 100 nM LCL161 (LCL), 20 μM z-VAD (Z), or 10 μM Nec-1 as indicated for 28 hours. **c**, **f**, **i**, Immunoblotting analysis of apoptosis (PARP and CC3) and necroptosis (pMLKL) markers in whole cell lysate of 22Rv1 (**c**), NCI-H358 (**f**), and BxPC-3 (**i**) cells treated as indicated for 20 hours.

D, DMSO; NCZ, nocodazole; PTX, paclitaxel; LCL, LCL161; Z, z-VAD. Cell viability was determined by measuring ATP levels. The data are represented as mean ± SEM of duplicate wells (**a**, **b**, **d**, **e**, **g**, and **h**). Results are reported from one representative experiment. Experiments were repeated independently for three (**a**, **b**, **d**, **e**, **g**, and **h**) or two (**c**, **f**, and **i**) times.

**Supplementary Fig. 9 | Combinatorial treatment of MTAs and Smac mimetics is efficacious in therapeutic intervention in breast cancer patient-derived xenografts (PDXs).**

**a**, **c**, **e**, and **g**, Effect of PTX and LCL161 (LCL) combinatory treatment on breast cancer PDX-2 (**a**), PDX-3 (**c**), PDX-4 (**e**), and PDX-5 (**g**). PDXs were developed and treated as described in Figure 7**c**, except tumor-bearing mice begin to receive treatment when tumor volume reached ~800 mm^3^. For PDX-2 study, n = 6 for each group. For PDX-3 study, n = 7 for Vehicle, PTX, and PTX/LCL groups; n = 6 for LCL group. For PDX-4 study, n = 8 for Vehicle group; n = 9 for PTX, LCL, and PTX/LCL groups. For PDX-5 study, n = 8 for each group. Scale bars, 2 cm. All graphs show mean ± SEM. p values for (**a**) and (**g**) were determined by the one-way ANOVA test, followed by Tukey’s multiple comparison post-test; p values for (**c**) and (**e**) were determined by the Kruskal-Wallis test, followed by Dunn’s multiple comparison post-test. NS, not significant; *p <0.05; **p <0.01; ***p <0.001. **b**, **d**, **f**, and **h**, 48 h (PDX-2) or 72 h (PDX-3, PDX-4, and PDX-5) after treatment as in Fig. 7**c**, tumors were isolated for histology (H&E staining) and apoptosis (cleaved caspase-3 IHC) analysis. Scale bars, 200 µm.

PTX, paclitaxel; LCL, LCL161. Results are reported from one representative experiment. Experiments were repeated independently for three (**c**) or two (**a**, **b**, and **d**-**h**) times.
