# Supplementary figures and images for: Membrane-bound TNF mediates microtubule-targeting chemotherapeutics-induced cancer cytolysis via juxtacrine inter-cancer-cell death signaling
Source: Cell Death Differ. 2019 Oct 23;27(5):1569–87. doi: 10.1038/s41418-019-0441-3 (PMC7206059; doi:10.1038/s41418-019-0441-3)

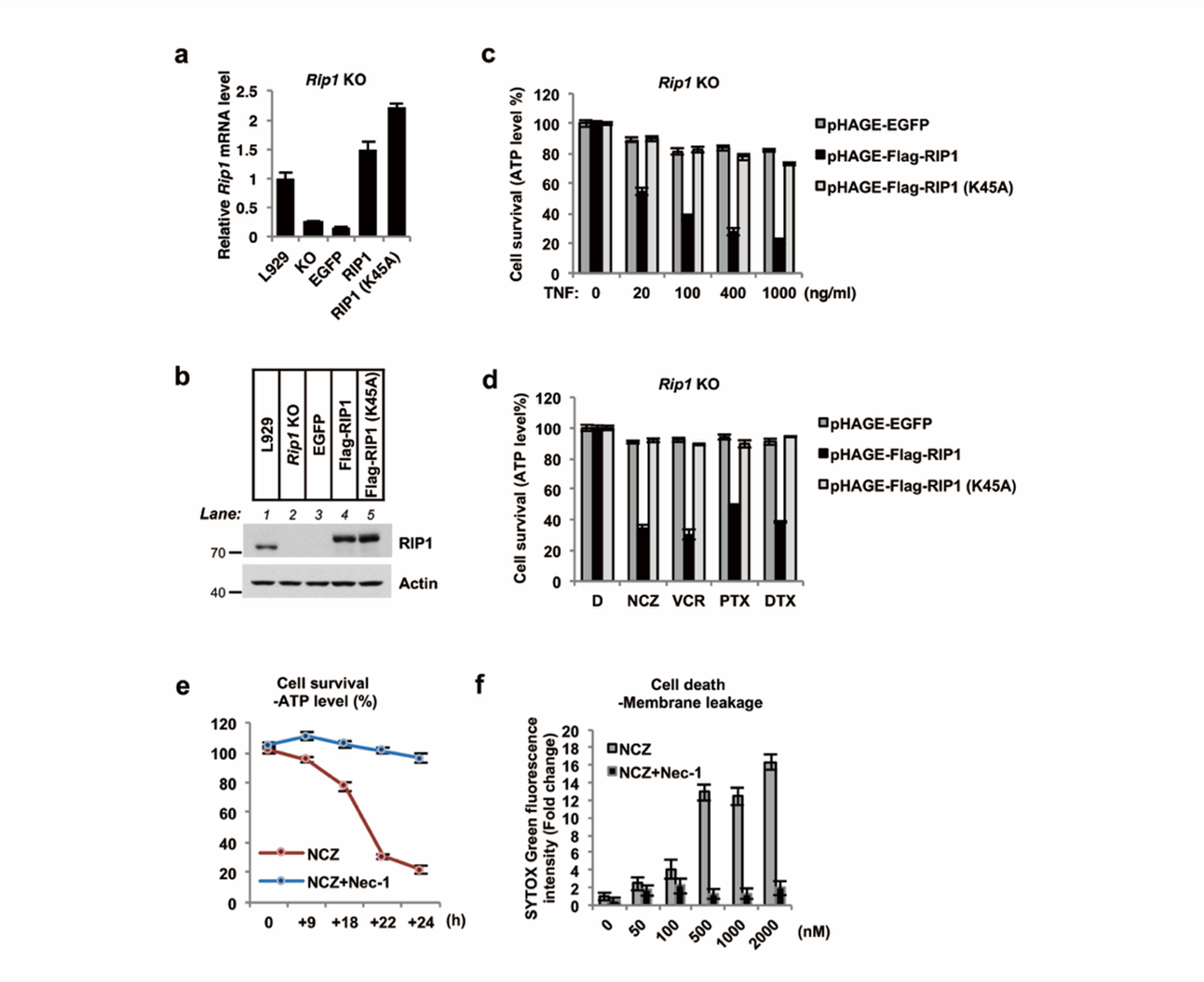

Supplement: Supplementary file 2 — Supplementary Fig. 1 [file 41418_2019_441_MOESM2_ESM.png]

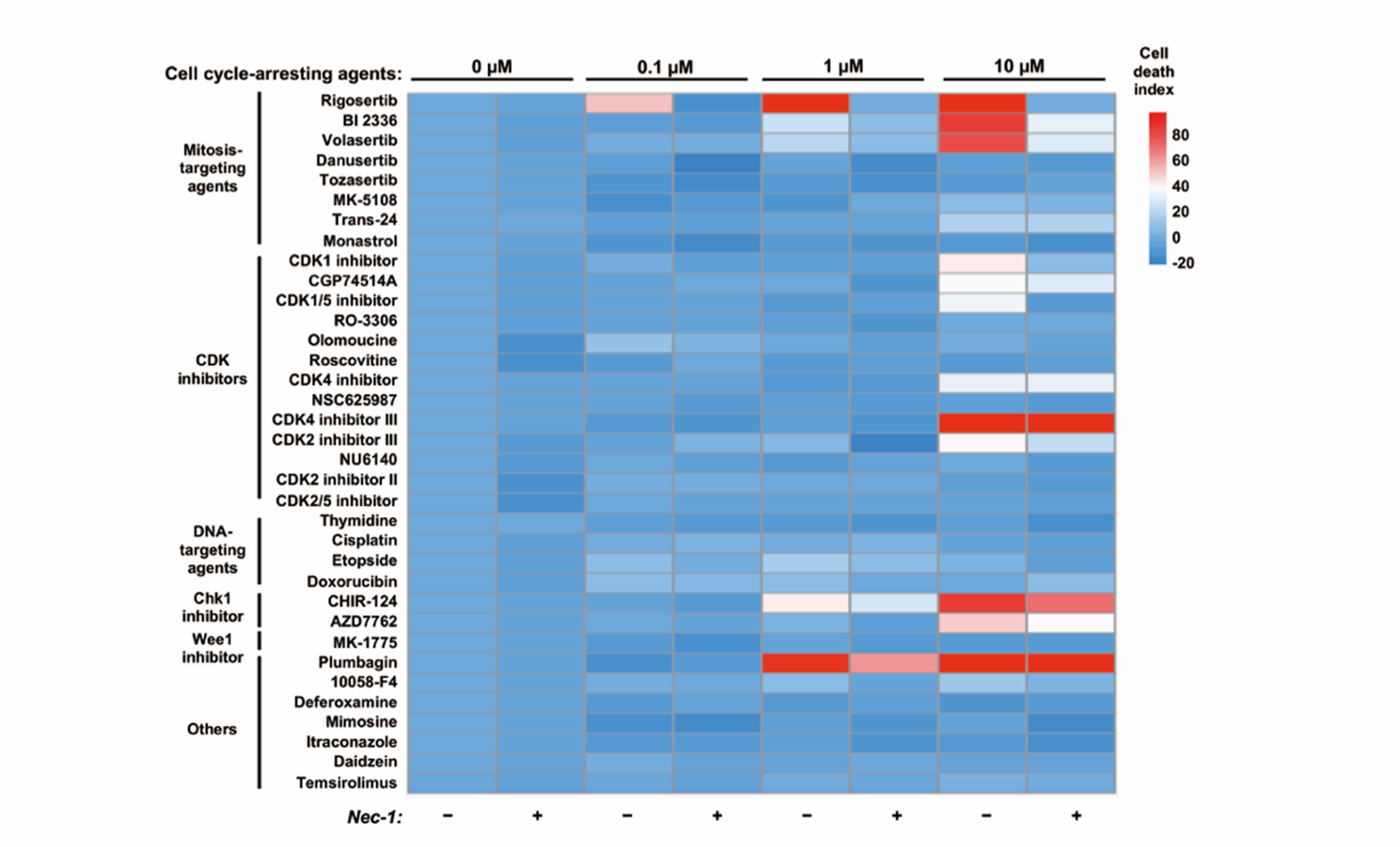

Supplement: Supplementary file 3 — Supplementary Fig. 2 [file 41418_2019_441_MOESM3_ESM.png]

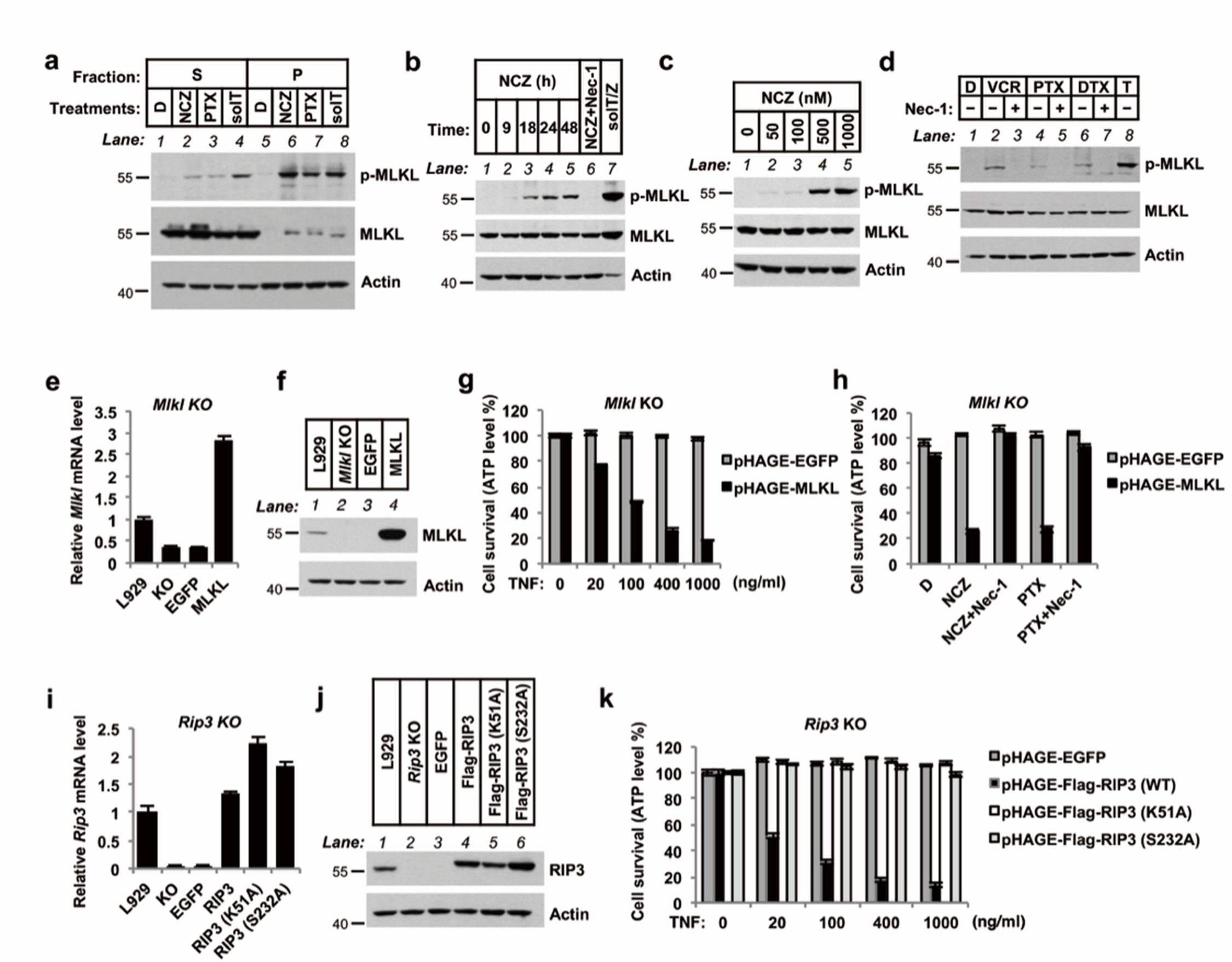

Supplement: Supplementary file 4 — Supplementary Fig. 3 [file 41418_2019_441_MOESM4_ESM.png]

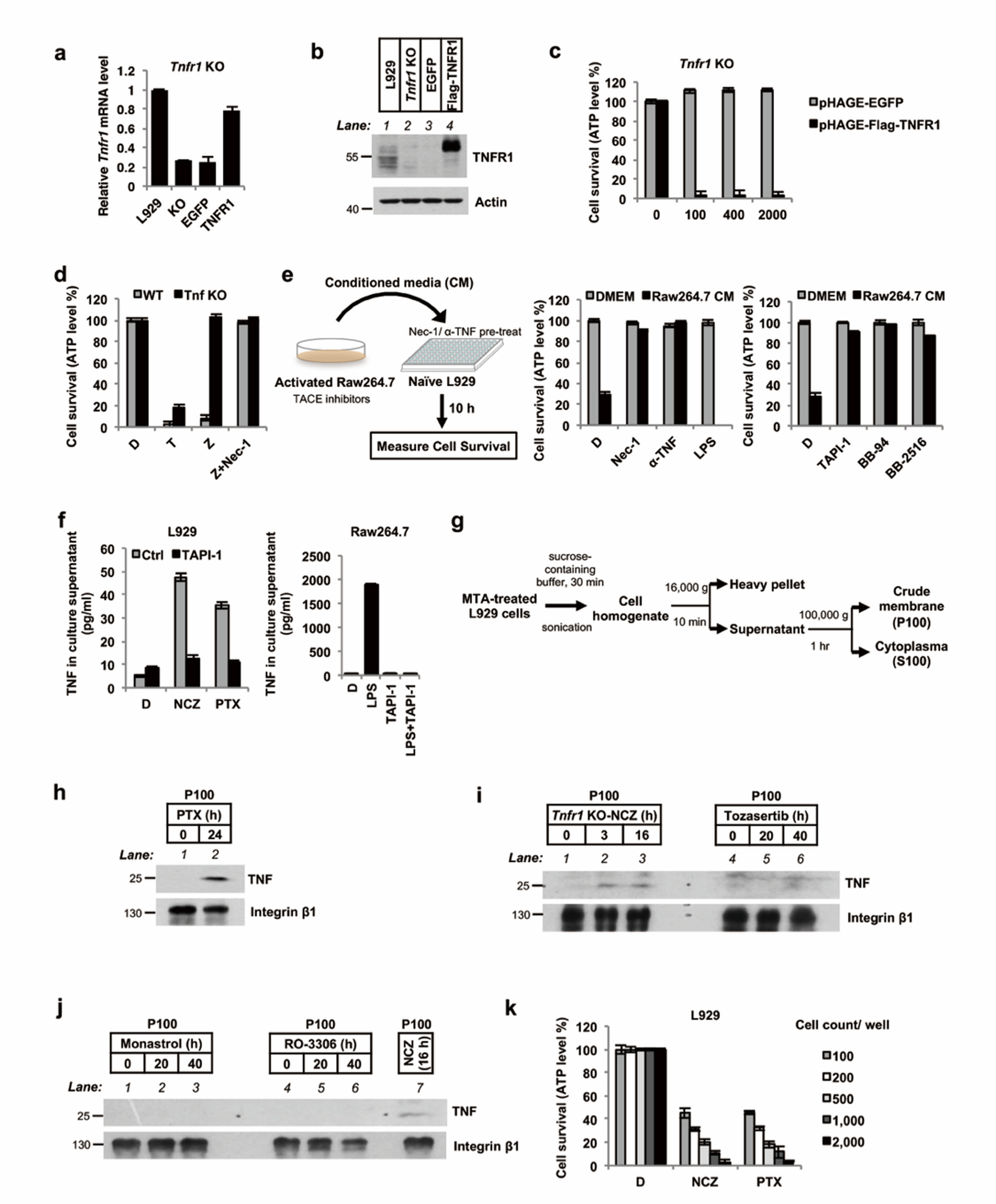

Supplement: Supplementary file 5 — Supplementary Fig. 4 [file 41418_2019_441_MOESM5_ESM.png]

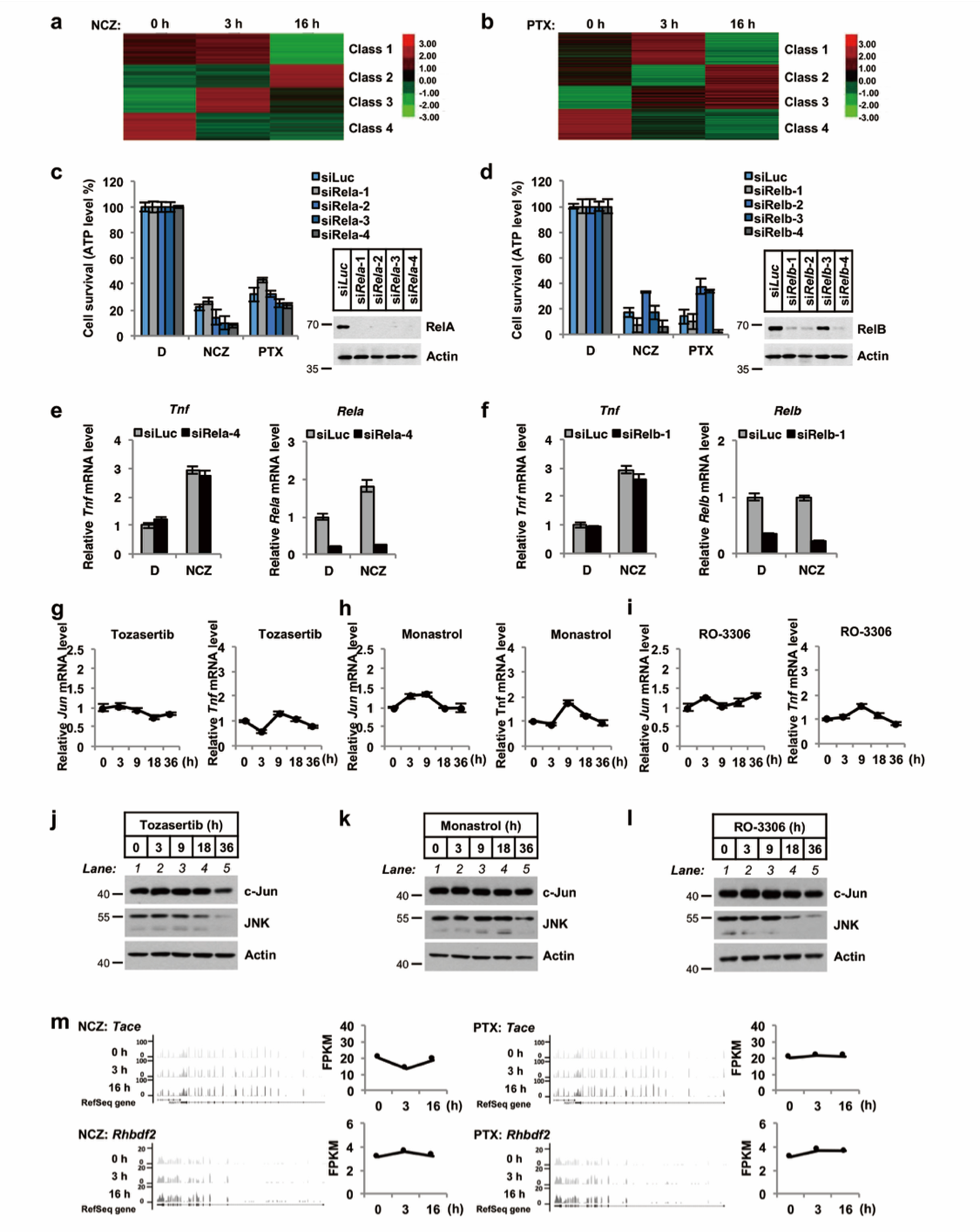

Supplement: Supplementary file 6 — Supplementary Fig. 5 [file 41418_2019_441_MOESM6_ESM.png]

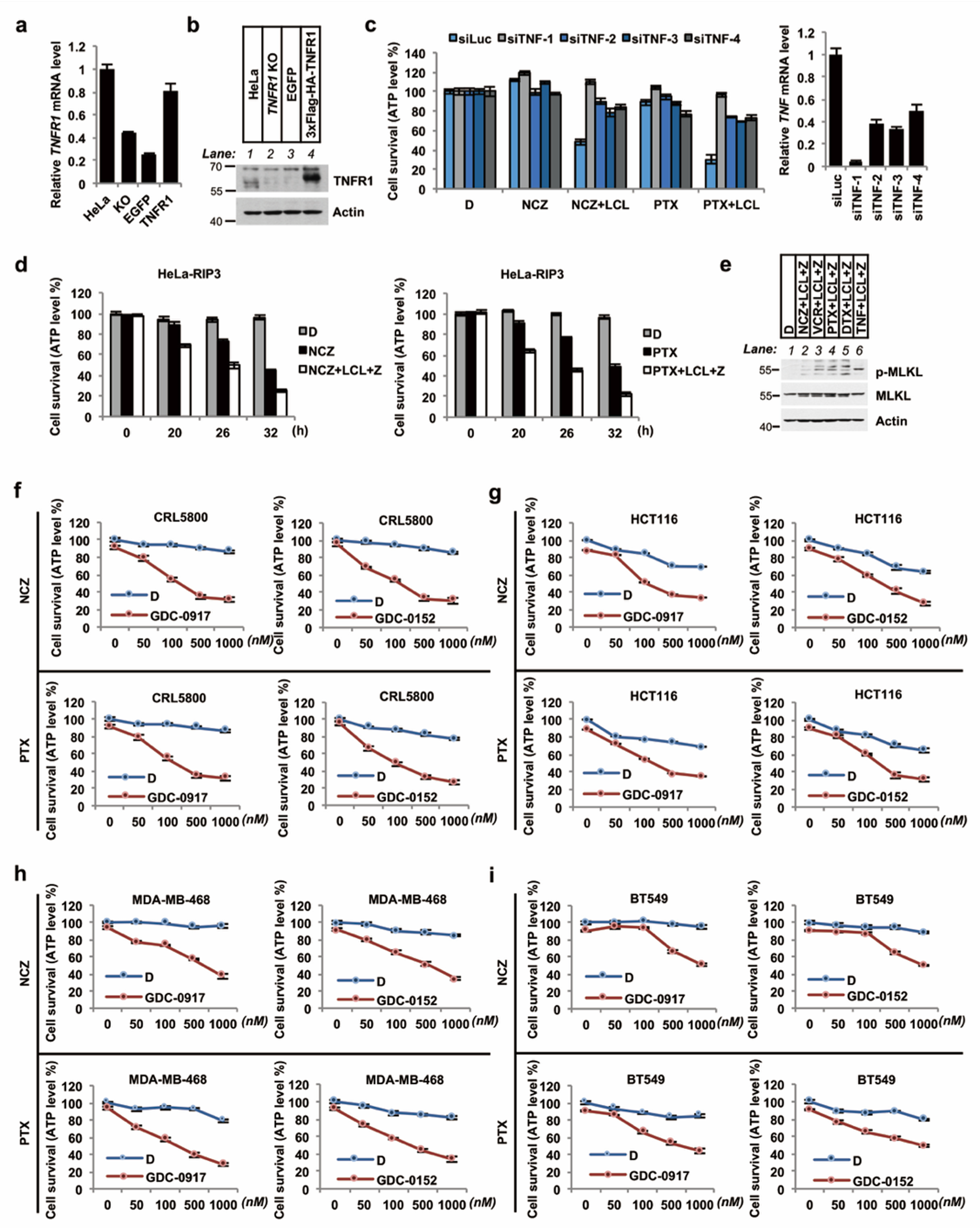

Supplement: Supplementary file 7 — Supplementary Fig. 6 [file 41418_2019_441_MOESM7_ESM.png]

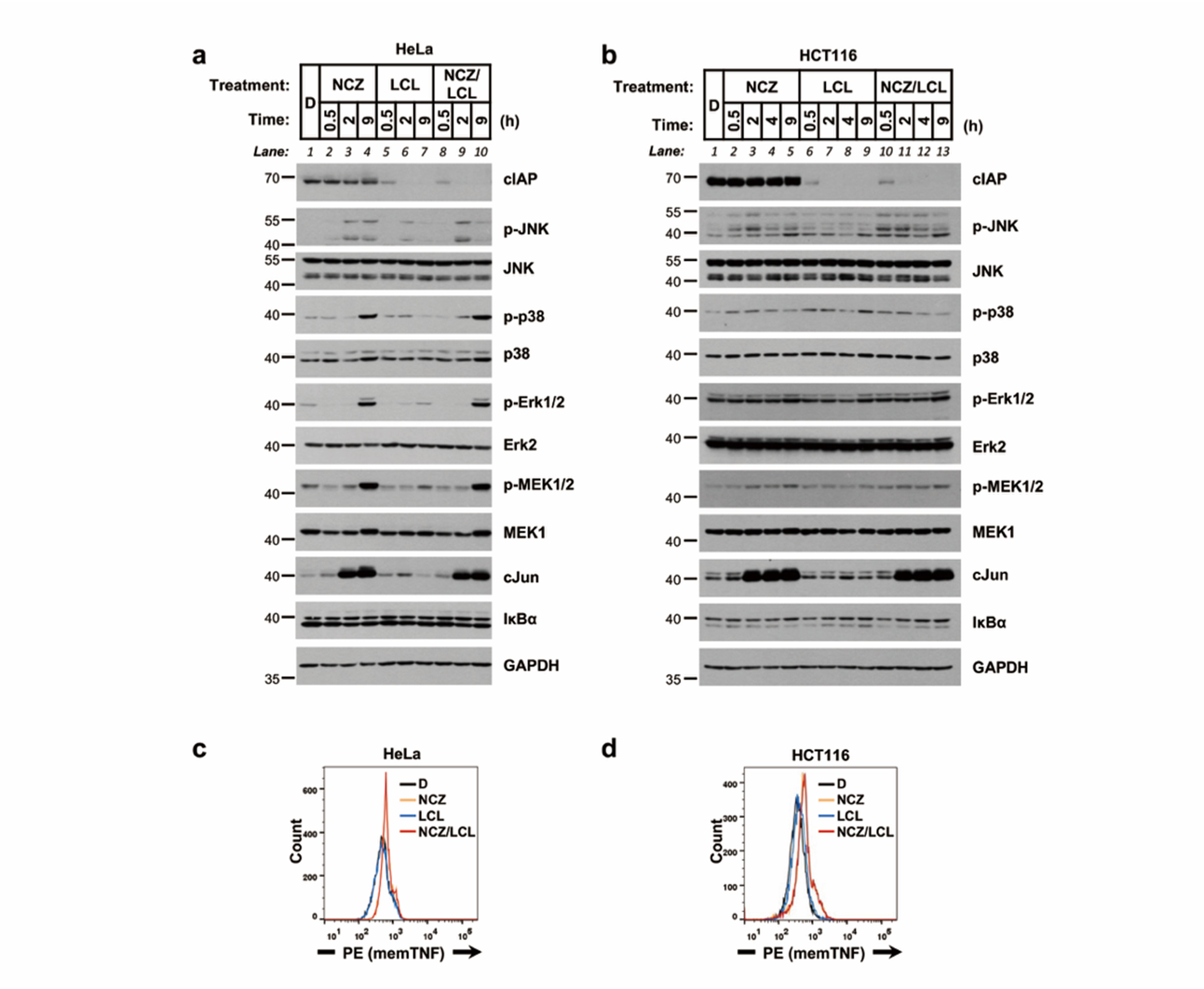

Supplement: Supplementary file 8 — Supplementary Fig. 7 [file 41418_2019_441_MOESM8_ESM.png]

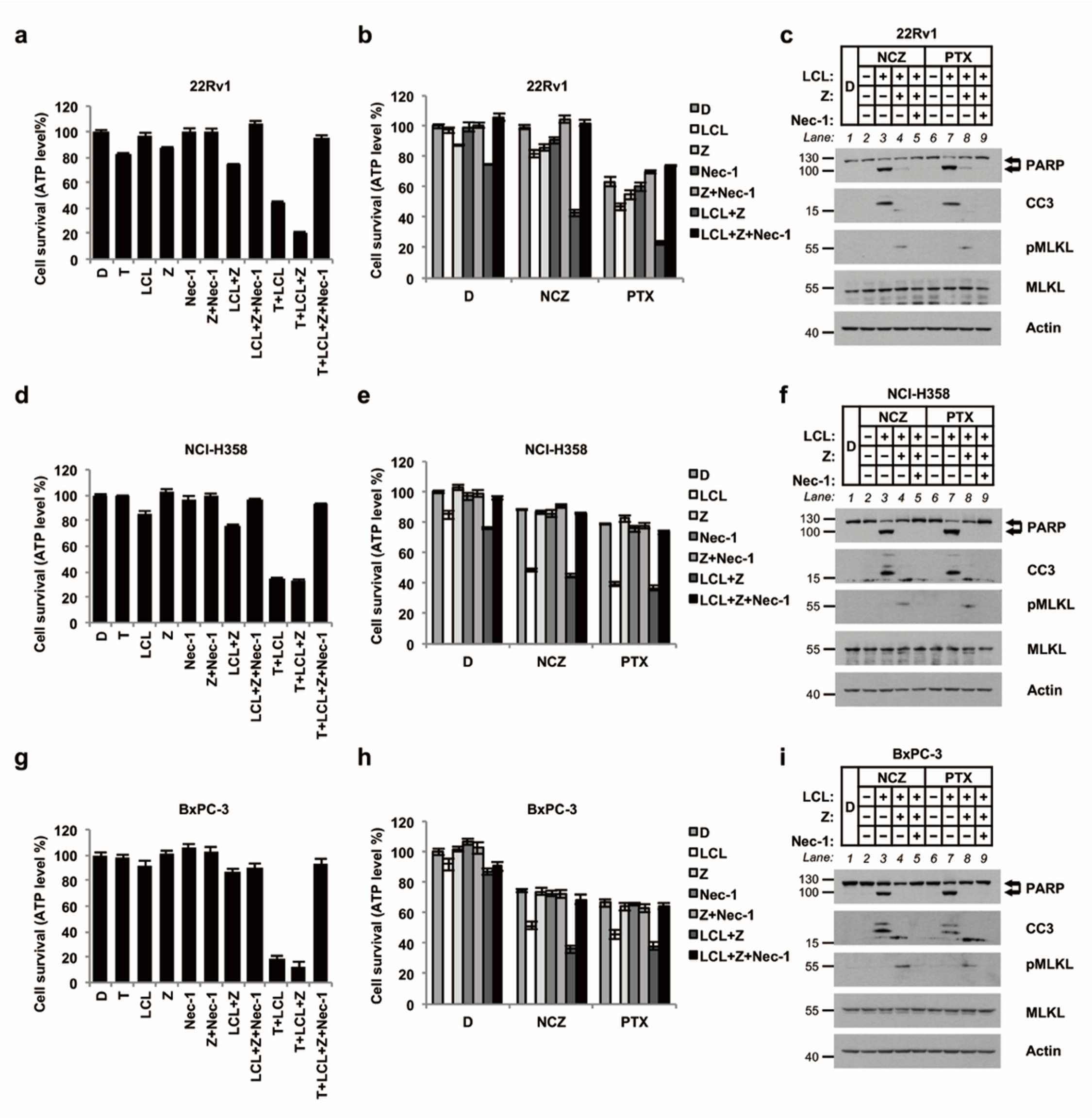

Supplement: Supplementary file 9 — Supplementary Fig. 8 [file 41418_2019_441_MOESM9_ESM.png]

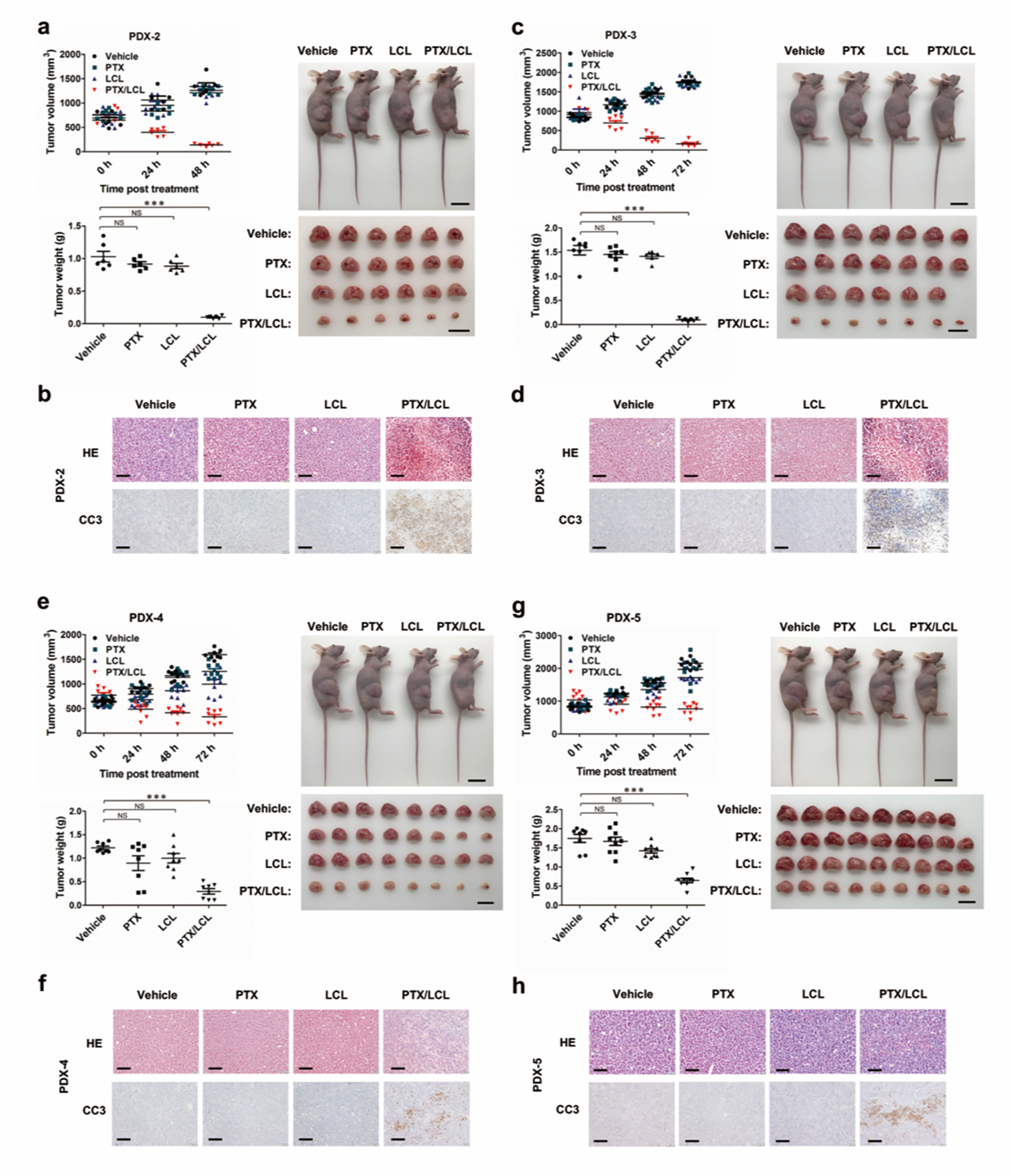

Supplement: Supplementary file 10 — Supplementary Fig. 9 [file 41418_2019_441_MOESM10_ESM.png]
