# Supplementary material for: Membrane-bound TNF mediates microtubule-targeting chemotherapeutics-induced cancer cytolysis via juxtacrine inter-cancer-cell death signaling
Source: Cell Death Differ. 2019 Oct 23;27(5):1569–87. doi: 10.1038/s41418-019-0441-3 (PMC7206059; doi:10.1038/s41418-019-0441-3)
Supplement: Supplementary file 11 — attribution of authorship [file 41418_2019_441_MOESM11_ESM.pdf]

DECLARATION OF CONTRIBUTIONS TO ARTICLE

ADMC

Manuscript Number: CDD-19-0868RR

Journal Name: *Cell Death & Differentiation* (the ‘Journal’)

Proposed Title of the Contribution: Author contributions (the ‘Contribution)

Author(s): Jing Zhang, Yu Yang, Shen’ao Zhou, Xueyan He, Xuan Cao, Chenlu Wu, Hong [illegible] (the ‘Authors’)

For all *CDD* articles, each person named as an author in the published version must be able to show he or she has contributed substantially to the article.

Authorship credit should be based on 1) substantial contributions to conception and design, acquisition of data, or analysis and interpretation of data; 2) drafting the article or revising it critically for important intellectual content; and 3) final approval of the version to be published. Authors should meet conditions 1, 2 and 3.

Any person who cannot be shown to have made a substantial contribution to the article cannot be listed as an author in the final version. The name of any person who is deemed to have made a minor contribution can, however, appear in the Acknowledgments section of the article.

Please complete the table below to indicate the contributions of all named authors to the manuscript.

Author Full Name:	Specification of Contribution to the Manuscript:
Jing Zhang	designed and performed the majority of experiments, analysed the data, and wrote the manuscript
Yu Yang	performed P100 fractionation assay and drew schematics
Shen'ao Zhou	constructed all the plasmids
Xueyan He	provided technical help on PDX study
Xuan Cao	performed bio-informatics analysis
Chenlu Wu	generated HeLa TNFR1 KO cells and helped with PDX study
Hong Hu	generated L929 Rip1 KO cells
Jie Qin	helped with the L929 fibrosarcoma allograft in nude mice and in vivo compound toxicity experiments in C57BL/6 mice
Gang Wei	conceived bio-informatics analysis
Huayi Wang	conceived the generation of L929 Rip1 KO cells
Suling Liu	contributed to the PDX materials; edited, reviewed and approved the final manuscript
Liming Sun	conceived the project, analysed the data and wrote the manuscript

Please complete the table below to indicate the contributions of all named authors to the figures.

Figure 1:

Jing Zhang, Shen'ao Zhou, Jie Qin, Liming Sun

Figure 2:

Jing Zhang, Yu Yang, Liming Sun

Figure 3:

Jing Zhang, Yu Yang, Shen'ao Zhou, Xuan Cao, Gang Wei, Liming Sun

Figure 4:

Jing Zhang, Shen'ao Zhou, Chenlu Wu, Liming Sun

Figure 5:

Jing Zhang, Shen'ao Zhou, Chenlu Wu, Liming Sun

Figure 6:

Jing Zhang, Liming Sun

Figure 7:

Jing Zhang, Yu Yang, Xueyan He, Chenlu Wu, Jie Qin, Suling Liu, Liming Sun

Signed for and on behalf of the Author(s):	Print Name:	Date:
	Liming Sun	2019/09/24
